# Hierarchical consciousness: the Nested Observer Windows model

**DOI:** 10.1093/nc/niae010

**Published:** 2024-03-18

**Authors:** Justin Riddle, Jonathan W Schooler

**Affiliations:** Department of Psychology, Florida State University, 1107 W Call St, Tallahassee, FL 32304, USA; Department of Psychological & Brain Sciences, University of California, Santa Barbara, Psychological & Brain Sciences, Santa Barbara, CA 93106, USA

**Keywords:** cognition, coherence, consciousness, cross-frequency coupling, hierarchy, information integration, neural oscillations, spatiotemporal scales, synchrony

## Abstract

Foremost in our experience is the intuition that we possess a unified conscious experience. However, many observations run counter to this intuition: we experience paralyzing indecision when faced with two appealing behavioral choices, we simultaneously hold contradictory beliefs, and the content of our thought is often characterized by an internal debate. Here, we propose the Nested Observer Windows (NOW) Model, a framework for hierarchical consciousness wherein information processed across many spatiotemporal scales of the brain feeds into subjective experience. The model likens the mind to a hierarchy of nested mosaic tiles—where an image is composed of mosaic tiles, and each of these tiles is itself an image composed of mosaic tiles. Unitary consciousness exists at the apex of this nested hierarchy where perceptual constructs become fully integrated and complex behaviors are initiated via abstract commands. We define an observer window as a spatially and temporally constrained system within which information is integrated, e.g. in functional brain regions and neurons. Three principles from the signal analysis of electrical activity describe the nested hierarchy and generate testable predictions. First, nested observer windows disseminate information across spatiotemporal scales with cross-frequency coupling. Second, observer windows are characterized by a high degree of internal synchrony (with zero phase lag). Third, observer windows at the same spatiotemporal level share information with each other through coherence (with non-zero phase lag). The theoretical framework of the NOW Model accounts for a wide range of subjective experiences and a novel approach for integrating prominent theories of consciousness.

## The case for a nested hierarchy

“We know what it is to get out of bed on a freezing morning in a room without a fire, and how the very vital principle within us protests against the ordeal. Probably most persons have lain on certain mornings for an hour at a time unable to brace themselves to the resolve. We think how late we shall be, how the duties of the day will suffer; we say, ‘I must get up, this is ignominious,’ etc.; but still the warm couch feels too delicious, the cold outside too cruel, and resolution faints away and postpones itself again and again just as it seemed on the verge of bursting the resistance and passing over into the decisive act.”

– William James, Principles of Psychology

William James eloquently captured the curious state of our mind in which competing cognitive streams coexist within consciousness: the desire to stay in a warm bed versus the responsibilities of the day (James 1890). Foremost in our experience is the intuition that we possess a unified conscious experience. However, many observations run counter to this intuition: we experience paralyzing indecision when faced with two appealing behavioral choices, we simultaneously hold contradictory beliefs, and the content of our thought is often characterized by an internal debate (Schwartz and Sweezy 2019). Thus, a perennial challenge to understanding the mind is conceptualizing how it accommodates a vast set of independent channels of information, while simultaneously generating a seemingly singular integrated “theater” of experience (Baars 1997). Although numerous empirical and theoretical advances helped conceptualize this issue (Baars 2005, Tononi et al. 2016, Mashour et al. 2020), the disparity of accounts illustrates its continued challenge. For example, one well regarded theory suggests that independent streams of information processing are integrated in a “global workspace” of consciousness (Baars 2005), whereas another theory argues this common “theater” of experience is an illusion and there are many coexisting narratives for the unfolding of events (Dennett 1988). Here, we propose the Nested Observer Windows (NOW) Model that unified consciousness, a single theater, exists at the apex of a vast nested hierarchy with control mechanisms to selectively attend to the underlying levels. Within the nested hierarchy, each spatiotemporal levels exhibits substantial autonomy with many observer windows at each level generating unique cognitive streams. The mind is akin to a supervisor observering detailed representations of sensory experience, initiating behavioral tasks with abstract commands, and resolving conflicting thoughts. The NOW Model addresses the complexity of subjective experience and generates testable predictions for cognitive neuroscience.

### Metaphors of mind

In order to capture ideas at the level of abstraction necessary to describe hierarchical consciousness and eventually to interrelate distinct theories, it can be helpful to draw on metaphors. By virtue of their incomplete mapping to their target, metaphors offer core scaffolding that can be further developed in different ways. Here we draw heavily on several metaphors of the mind, some fresh others well worn, to characterize a general framework for how the mind may integrate numerous streams of information into a seemingly holistic experience.

**Figure 1.**
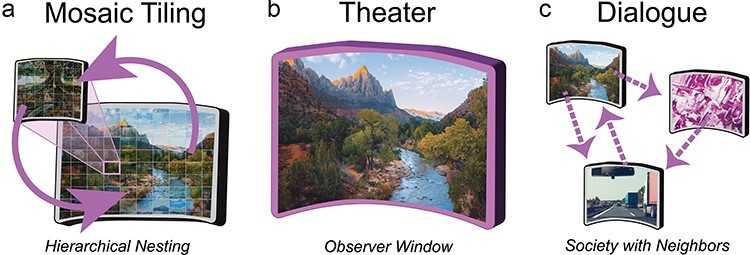
Central metaphors of the NOW Model. (A) MOSAIC TILING: Observer windows are nested within each other across spatiotemporal scales like a mosaic composed of mosaics. Each observer window gathers information from its subwindows. (B) THEATER: Each observer window is a theatre that binds information into a single gestalt representation. Spatial and temporal boundaries constrain what information is integrated within an observer window. The processing speed of an observer window is proportional to its size and scale. (C) DIALOGUE: Observer windows engage in dialogue to resolve conflicting viewpoints and acquire information from each other. Collectively, observer windows interact constructively to form a society or corporation. The central component of each metaphor is emphasized in purple.

*Mosaic tiling metaphor*: The defining property of the NOW Model which sets it apart from many theories of consciousness is that observer windows are nested within each other such that the information is shared vertically across spatiotemporal scales, e.g. localized electrical activity in place cells within the hippocampus and the electric fields that engulf the hippocampus (Lisman and Jensen 2013). By comparison, most theories of consciousness propose a single spatiotemporal level at which consciousness is created, typically at the level of neurons (Lau and Rosenthal 2011, Tononi et al. 2016, Mashour et al. 2020), but see (Northoff and Huang 2017). Nested observer windows are likened to mosaic tiling where an image is composed of mosaic tiles, each of these tiles is itself an image (Fig. 1a). The relationship between tiles and gestalts occurs at multiple levels with each individual tile representing a gestalt of still smaller tiles, which in turn are gestalts of yet smaller tiles, and so on. In the NOW Model, nested mosaic tiling generates abstraction through bottom-up signaling, i.e. emergence, and abstract intentions or interpretations are translated into actionable motor commands or constraints on processing via top-down signaling, i.e. submergence. Although embedded in a hierarchical structure, each observer window maintains its own gestalt representation, or theater. Therefore, the NOW Model asserts the presence of multiple cognitive streams within a single brain, not only in a given spatiotemporal scale, but at many spatiotemporal scales. Critically, the NOW Model proposes that unitary consciousness resides at the apex of the hierarchy, and simultaneously allows for separate cognitive streams to reside at deeper layers of the nested hierarchy.

*Theater metaphor*: Ever since the incisive analysis by Dennett and Kinsbourne (Dennett and Kinsbourne 1992), scholars are warry of referring to consciousness as a theater where it all comes together. Nevertheless, there is no mistaking the fact that conscious experience *feels* very much like a theater, which is probably why theaters so effectively sweep us up in their drama. Regardless of its ultimate ontological reality, the theater metaphor compellingly captures the phenomenal experience of consciousness as it unfolds. In the NOW Model, we propose that observer windows are the sole location for the integration of information. Like a theater, observer windows are constrained by definitive boundaries and with a characteristic speed at which events unfold, referred to as its processing speed (Fig. 1b). Many observer windows exist within the brain at different scales, e.g. cortex and neurons, and these observer windows are fundamentally independent from each other and only loosely interact. Thus, a fundamental implication of the NOW Model is that multiple independent cognitive streams coexist within the brain. In the “Mosaic tiling with cross-frequency coupling” section, we discuss the potential for every observer window versus a subset to correspond with a conscious experience versus a non-conscious cognitive stream.

*Dialogue metaphor*: A number of scholars have used a “society” (Minsky 1988) or “corporation” (Loftus and Schooler 1985) as a metaphor for the mind. Like corporations, minds engage in multiple tasks simultaneously and their efficacy depends on the distribution of responsibility across multiple independent modules (Fodor 1983). In the NOW Model, each observer window operates independently and is likened to an individual in a corporation. Here, a sharp contrast is drawn between a theater of experience which binds information into a holistic representation and a dialogue between observer windows for the purpose of sharing information (Fig. 1c). Cognitive dissonance arises from a breakdown in effective communication.

It is often said that one should avoid mixing metaphors as doing so can lead to confusion. However, here we take the opposite tack, arguing that using multiple metaphors is actually advantageous as each metaphor can be particularly effective in illuminating different aspects of the overall framework. However, aware of the risks of confusion, we will attempt to map the metaphors on to one another whenever possible.

### Quantification in the NOW model

**Figure 2.**
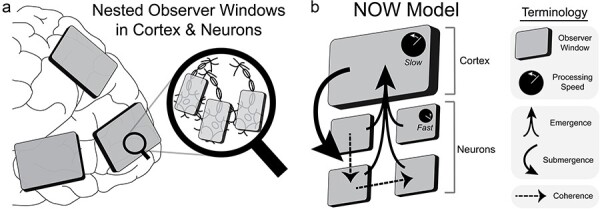
Applying the NOW Model to the brain. (A) Evidence from cognitive neuroscience supports the NOW Model in cortex and neurons. (B) The NOW Model is a conceptual framework for investigating the neural basis of cognition from a minimal set of principles. Cross-frequency coupling provides a means of emergent and submergent signaling. Synchrony defines observer windows with a processing speed corresponding to its spatiotemporal scale. Coherence enables dialogue between observer windows that are within the same spatiotemporal level

For each metaphor in the NOW Model, we provide a description of its presentation to the tools of cognitive neuroscience with a corresponding means of quantifying its presence in electrical activity, e.g. in cortex and in neurons (Fig. 2a). The three principles of the NOW Model are (i) emergence/submergence of signals between nested observer windows using cross-frequency coupling, (ii) the definition of an observer window with synchrony, and (iii) dialogue between observer windows via coherence (Fig. 2b). The critical role of electrical activity to cognitive processing in the brain is appreciated by scholars (Singer and Gray 1995, Fries 2005, Canolty and Knight 2010, Levin and Martyniuk 2018) and electric fields are proposed by some to be the substrate for consciousness (Nunez and Srinivasan 2006, Hunt and Schooler 2019). However, we remain agnostic as to the specific algorithm, or mechanism, that processes information as the signals discussed here may be necessary but not sufficient to generate consciousness (Box 1). Nonetheless, these three principles correspond to specific physiological signals that were causally validated using brain stimulation as serving a mechanistic role in cognition through experimentation (Sauseng et al. 2009, Alagapan et al. 2019, Riddle et al. 2020a, 2021). These causal tests for the three principles are elaborated in the following three sections. From this basis, the NOW Model generates testable predictions as to the nature of consciousness.

As a whole, we use the acronym “NOW” to refer to the independent occurrence of a multitude of processing speeds across many nested spatiotemporal scales. Each observer window undergoes successive moments of “now” at its own speed and yet the contents of its theater contain representations from lower-order observer windows with a notably faster speed of processing. Hence, the relatively slow processing speed at the apex is able to integrate over many sources of faster activity a level beneath in cortex. In the following three sections, we provide evidence for each principle, its contribution to cognition, and potential implications for conscious experience. Finally, in “The apex observer window” section, we propose that subjective experience as we know it likely resides at the apex of the observer window hierarchy.

## Mosaic tiling with cross-frequency coupling

Box 1. **Quantification in the NOW Model**

Observer Windows are identified in the power spectrum of Fourier-decomposed electrical activity as a Gaussian distribution (*G*) superimposed on background noise (*L*). Gaussian: $G = h*\exp \left( {\frac{{ - {{\left( {F - c} \right)}^2}}}{{2{w^2}}}} \right){\text{ }}$, *h* is height, *w* is width, *c* is the peak frequency/processing speed. *F* is frequency. Background noise: $L = b - \log \left( {{F^x}} \right){\text{ }}$, *b* is the estimated intercept, *x* is slope of noise distribution.

Cross-frequency coupling of NOW at neighboring spatiotemporal scales is measured using phase-amplitude coupling (PAC): the phase (θ) of a low-frequency observer window (A) is coupled to the amplitude (M) of a high-frequency observer window (B). $PAC = \left| {\frac{{\mathop \sum \nolimits_{t = 1}^N {M_A}*{e^{i{\theta _B}}}}}{N}} \right|$

*Synchrony* is a strongly coupled system quantified by a zero phase lag relationship between internal signals, *C* and *D*, with matched processing speed and a positive correlation coefficient: *r*  $ = {\text{ }}\frac{{\mathop \sum \nolimits_{t = 1}^N\left( {{C_t} - {\mu _C}} \right)\left( {{D_t} - {\mu _D}} \right)}}{{\sqrt {\mathop \sum_{t = 1}^N{{\left( {{C_t} - {\mu _C}} \right)}^2}{\text{ }}\mathop \sum \nolimits_{t = 1}^{N{{\left( {{D_t} - {\mu _D}} \right)}^2}}} }}$, μ is mean, *N* is number time points, *t* is time.

*Coherence* is transient alignment of two observer windows (E and F) with non-zero phase lag. Weighted-phase lag index (wPLI) is the magnitude of the sum of the imaginary component of the cross-spectral density of two signals. $\mathcal{H}{\text{ }}$is the Hilbert transform and assumes that *E* and *F* are band-limited signals with nearly the same processing speed, i.e. at the same spatiotemporal level. $wPLI = \left| {\mathop \sum \limits_{t = 1}^N {\text{imag}}\left( {{\text{ }}\mathcal{H}\left( E \right)*\overline {\mathcal{H}\left( F \right)} {\text{ }}} \right)} \right|$, “*imag”* takes the imaginary component.

We feel a sense of ownership over our thoughts and take pride in our capacity for creative imagination. However, thoughts often spring into our mind fully formed without any apparent point of origin (Gable et al. 2019). People are sometimes presented with unwanted thoughts that are not generated of their own volition and these thoughts are willfully inhibited (Wegner 1992). At other times, we do have the experience of intentionally assembling a complex gestalt perception from a few component concepts; but this manipulation occurs at a high level of abstraction, as in hierarchical cognitive control tasks (Collins and Frank 2013, Badre and Nee 2018). Furthermore, the intention to act is initiated at an abstract level that subsequently generates an intricate series of muscle movements; and yet, we do not require knowledge of these muscles, and typically possess no awareness of the activation of each set of muscles (Wegner and Wheatley 1999). In the NOW Model, our capacity to perceive and to act exists at an abstract level encompassing the details, but not burdened by them, with a limited interface to the underlying levels.

### Abstraction and top-down control

When a person swats at a house fly, the fly is easily able to dodge the attack despite the relatively greater level of intelligence of the human. The decision to swat a fly, the initiation of motor action, and the muscle innervation is slow relative to the rapid perceptual integration and decision processes of the fly. When watching a fly explore its environment, the fly appears to teleport into different bodily orientations without any apparent fluid motion. The processing speed of the perceptual and motor observer windows of the fly are orders of magnitude faster than those of the human; and, therefore, the fluid movement of the fly is imperceptible to human visual processing (Umeton et al. 2017). Evolution selected for a slower perceptual system in humans despite the advantage of avoiding predation with a faster system (Gray et al. 2003). The eyes of a fly and the eyes of a human do not differ significantly; however, the primate brain is able to generate representations of exceeding complexity and extract many levels of abstraction beyond the trivial detection of light at different wavelengths (Bruce et al. 1981). The complexity of perceptual and decision-making processes in the human are orders of magnitude more nuanced and abstract: contexts are considered, repercussions are simulated, temporally delayed results are entertained, and metaphoric significance is comprehended.

**Figure 3.**
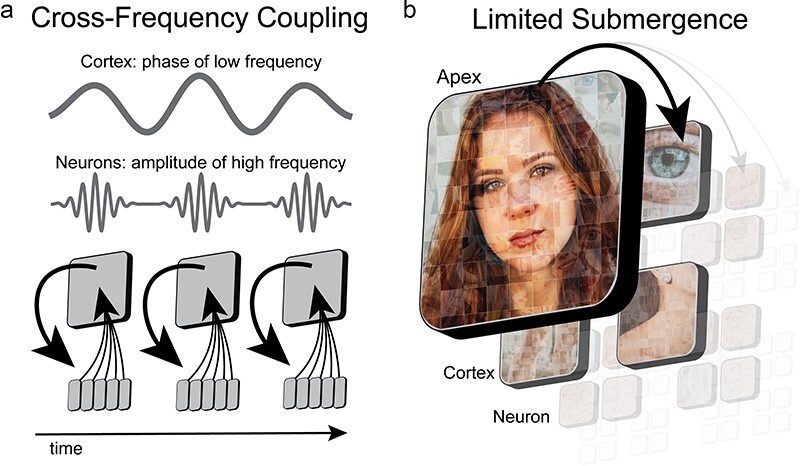
Mosaic tiling with cross-frequency coupling. (A) Cross-frequency coupling between observer windows at different spatiotemporal scales is a weak form of coupling. For example, the phase of low-frequency activity in cortex couples to the amplitude of high-frequency activity in neurons. (B) With weak coupling, observer windows are limited in their access and influence over nested observer windows. Limited influence from the apex observer window is depicted as progressively increased opacity for each lower level and for the arrow toward it

Within the NOW Model, the bottom-up feed of information from increasingly smaller and faster observer windows is abstracted through cross-frequency coupling by larger and slower observer windows (Jensen and Colgin 2007, Canolty and Knight 2010, Palva and Palva 2018) (Fig. 3a). An early observation of cross-frequency coupling was between theta oscillations in the hippocampus that encoded a trajectory of movement and high-frequency activity of its neurons that encoded specific locations in space (O’Keefe and Recce 1993, Brun et al. 2002, Colgin et al. 2009, Lisman and Jensen 2013, Agarwal et al. 2014). Critically, motion is a sequence of places, not the binding of multiple places into a more detailed place. A distinction is drawn between *binding* implemented via synchronization (see “The cognitive theater: binding of experience by synchrony” section) and *abstracting* implemented via cross-frequency coupling. Binding is the integration of features into a more *detailed* representation that encompasses all of the individual features, whereas abstraction is an emergent property where the level of description is transformed into a higher-order representation from a lower-order representation through sequencing, ordering, or some other yet to be determined process. Human subjective experience at the apex of the nested observer windows hierarchy is limited to a relatively narrow and slow frequency range, but contains the richness of information processing from hundreds of cortical regions and from billions of neurons (and trillions of proteins) that integrate information at faster timescales. When information is abstracted into a higher level, the details of the lower levels are packaged into a gestalt such that we experience a rich perceptual environment and yet we are able to navigate this environment using a reduced set of icons. This framing is similar to Donald Hoffman’s interface theory of perception, in which subjective experience is akin to a user interface (D. D. Hoffman et al. 2015), but in the NOW Model this interface is abstracted through the nested hierarchy.

Humans have the experience of controlling their body (Wegner and Wheatley 1999, but see Metzinger 2017). The decision to perform a given action occurs at a relatively slow rate, on the order of a couple decisions per second (Wyart et al. 2012), yet the repercussions of these simple commands involve complex patterns of muscle innervation and visuomotor coordination. While a perceptual observer window generates abstraction from smaller-faster observer windows through emergence, a goal-directed observer window in frontal cortex initiates an action plan that is propagated down to its subsystems through submergence (Voytek et al. 2015a, Helfrich et al. 2017, Fiebelkorn et al. 2018, Riddle et al. 2021). As a professional pianist initiates a cartoonish higher-order template for action, this abstract command submerges from a higher-order observer window into the motor system and carries out a fluid motion trained by thousands of hours of experience (O’shea’s and Moran 2018). The NOW Model provides a ready explanation for the simplicity of subjective human motor commands as compared to the complexity of bodily action. The lower-level observer windows act in a semi-autonomous fashion allowing for learning to occur at spatiotemporal scales removed from subjective awareness, e.g. motor neurons in the spinal cord. The NOW Model posits that action commands can be generated at the macroscopic spatiotemporal scale of functional brain regions and these action commands submerge into the smaller and faster scales via cross-frequency coupling.

Certain cross-frequency coupling pairs such as delta (2–4 Hz) to beta (15–30 Hz) coupling may be particularly relevant for guiding top-down goal-directed behavior, whereas theta (4–8 Hz) to gamma (50–200 Hz) coupling may be particularly relevant for bottom-up perceptual processing (Morillon et al. 2019, Riddle et al. 2020b, 2021, 2022). Delta–beta coupling might be characterized as the translation of simple action commands from the executive-control network into activation of neurons in the motor cortex and muscles, whereas theta–gamma coupling translates perceptual features from visual and auditory cortex into a greater context within the hippocampal memory network during long-term memory formation (Hermiller et al. 2020). Beyond correlational evidence, brain stimulation was delivered to mimic the cross-frequency coupling activity patterns observed between cortex and neurons. For example, delivery of theta–gamma cross-frequency transcranial alternating current stimulation (tACS) improved working memory performance when delivered to prefrontal cortex more so than theta-frequency tACS or gamma-frequency tACS on their own (Alekseichuk et al. 2016). Cross-frequency tACS was also demonstrated to differentially engage the motor preparation system when delivered to mimic delta–beta coupling versus memory access with theta–gamma coupling (Riddle et al. 2021). These studies provide evidence that cross-frequency coupling serves a causal role in translating higher-order cognitive functions in cortical observer windows to observer windows in neurons.

### Capacity limits on control and attention

The nested hierarchical organization of observer windows is distinct from the classical description of a hierarchy. Classical hierarchies describe a supervening system that exerts unidirectional influence over one or more systems within the same spatiotemporal scale (Felleman and Van Essen 1991, Riesenhuber and Poggio 1999, Luria 2012, Murray et al. 2014, Taylor et al. 2015). In contrast, the integration of information within the nested hierarchy of the NOW Model more closely resembles mosaic tiling in which a gestalt representation is formed out of the binding of lower order representations. Similar to coherence, cross-frequency coupling is a weak form of coupling that retains the autonomy of lower-order NOW. This property results in a fundamental limitation for the observer window to exert influence (top-down) and a limitation to comprehend the vast quanitites of incoming information (bottom-up) (Fig. 3b).

For example, despite understanding that a percept violates common sense, e.g. the illusion of an undulating checkered pattern, the perception of the optical illusion is unavoidable. This example highlights that top-down control signals are unable to meaningfully alter the information processed by the lower-order observer windows generating the optical illusion. On the contrary, top-down control signals were shown to be effective in flipping between bistable perceptions as with the Necker cube (Kornmeier et al. 2009). Here, a higher-order observer window changed the activity of a lower-order observer window presumable via cross-frequency coupling. Curiously, this ability is accentuated in long-time mediators (Kornmeier et al. 2017), correlated with creativity (Wiseman et al. 2011) and displays considerable individual differences in voluntary control (Sauer et al. 2012).

In a similar manner, the process of emergence (bottom-up transfer of information) sometimes results in larger observer windows receiving conflicting information. For example, in binocular rivalry two different images are presented briefly and simultaneously to each eye and the competing perceptual streams cannot be integrated (Tong et al. 2006). In this case, people are able to switch between paying attention to one percept or the other, but not to both. Thus, a primary goal of supervening observer windows is to resolve conflicting information between lower observer windows. Binocular rivalry also highlights a fundamental limit in attentional capacity. Vast quantities of data are presented and integrated into a single theater, including internally generated representations and abstract thought not tied to the external world. The rich subjective landscape is parsed by a spotlight of attention that fixates on some elements at the exclusion of others. Accordingly, the NOW Model predicts that cross-frequency coupling dictates which nested observer windows receive the focus of attention. During attention capture, observer windows can spring into the focus of attention, e.g. when absent-mindedly driving a car and traffic suddenly hits a stand still. Thus, the NOW Model presents a complex relationship between a supervening observer window and its subwindows where the information that is passed between levels can be determined by either level under certain circumstances: bottom-up attention capture and top-down selective attention.

### Nested consciousness

**Figure 4.**
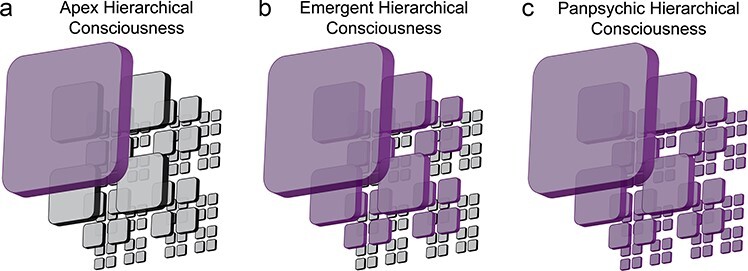
Consciousness in the NOW Model. Consciousness could emerge in the NOW Model in three distinct manners. (A) In apex hierarchical consciousness, only the observer window at the top of the hierarchy is conscious. (B) In emergent hierarchical consciousness, all observer windows above a threshold are conscious. The top three levels are depicted to be conscious, but this threshold could be applied at any level. (C) In panpsychic hierarchical consciousness, all observer windows possess a conscious experience. Purple denotes the presence of consciousness in an observer window

A curious proposition compatible with the NOW Model is that some, or all, of these nested observer windows possess a conscious experience of their own. In principle, there are three general possibilities. The most traditional account is what we term the *apex hierarchical consciousness* view, namely that consciousness exclusively arises at the scale of the largest slowest observer window, the apex (Fig. 4a). This window would correspond to the “global workspace” of global workspace models, e.g. (Dehaene et al. 1998, Baars 2005, Baars and Franklin 2007), that posits that consciousness exclusively arises when information across brain modules is integrated into a singular information stream (Baars 1997, 2005). Global neuronal workspace posits that frontal–parietal neurons are the substrate for the global workspace, whereas the NOW Model proposes a more general mechanism, e.g. layer 1 of the cerebral cortex (see “The apex observer window” section). The apex of the NOW Model is conceptually similar to Walter Freeman’s model of consciousness except that Freeman insisted on high-frequency global synchrony in the gamma band (Freeman 2015).

The other two classes of models involve consciousness arising in lower-level observer windows as well as the apex. In emergent hierarchical consciousness, only observer windows that achieve some critical level of informational complexity, or other yet to be determined criteria, are conscious (Fig. 4b). Such an account would correspond in a general sense to Zeki’s hierarchical model (Zeki 2003) that posits multiple independent streams of consciousness exist at three levels near the top of the biological hierarchy: unified consciousness (the apex), macro-consciousness (level beneath the apex), and micro-consciousness (two levels beneath the apex). Although there could be a variety of ways in which emergent hierarchical consciousness might be instantiated, the key notion is that distinct streams of consciousness are associated with multiple, but not all, observer windows.

A final way in which consciousness might pertain to the NOW Model entails the panpsychist claim that consciousness is a fundamental aspect of reality and, therefore, exists within all systems that integrate information (Chalmers 2015). In the NOW Model, panpsychic hierarchical consciousness is when every observer window at every spatiotemporal scale corresponds to a conscious entity (Fig. 4c). You just happen to be the consciousness at the top of the hierarchy. Such an account would be consistent with panpsychist theories of consciousness such as Hunt and Schooler (Hunt and Schooler 2019; J. Schooler 2014; J. W. Schooler et al. 2011a). However, other panpsychist theories such as Koch and Tononi (Tononi and Koch 2015) do not entail hierarchical consciousness. See Goff (2020) and Goff and Coleman (2020) for a discussion regarding the often fuzzy definition of the physical world and sub-categories within panpsychism. Critically, the NOW Model imposes limitations on panpsychic hierarchical consciousness by requiring at least zero-phase lag synchronization for the formation of an observer window and posits that this likely requires biological infrastructure, but may allow for transient proto-conscious experience at more fundamental physical scales.

### Testable predictions and open questions

- Goal-directed behavior and abstract thought originate in higher-order cortex in the form of low-frequency oscillations that then submerge into neural actvity. For example, in mind-wandering spontaneous thoughts originate in higher-order cortex without a bottom-up origin. Thus, electrical recordings of the brain should reveal the appearance of low-frequency activity followed by increased cross-frequency coupling with lower levels.
- While reading a book, a common experience is that of mind-wandering (J. W. Schooler et al. 2011b, Smallwood and Schooler 2015). As the mind wandered to relive an episodic memory, both eyes diligently moved from word to word and a hand flipped to an entirely new page (Reichle et al. 2010). However, no information content was integrated in higher-order observer windows (J. W. Schooler 2004). The NOW Model predicts that lower-order observers do not show cross-frequency coupling when their information content is not abstracted into a higher-order observer window.
- With cross-frequency coupling guiding interactions across spatiotemporal scales, recordings of the brain should find evidence of this nested hierarchy. In support of this model, a prominent 1/f power law, or pink noise, is evident in electrical recordings of the brain (Bedard et al. 2006, He 2014, Voytek et al. 2015b), heart beats (Ivanov et al. 1999), neuron spiking (Teich 1989), and protein surfaces (Goetze and Brickmann 1992). Future work should investigate how this activity changes under diffferent cognitive states.
- There are multiple types of cross-frequency coupling that were observed in brain activity: phase-amplitude coupling, phase–phase coupling, and amplitude–amplitue coupling. It is currently unclear whether these different forms of coupling correspond with different forms of information transfer across spatiotemporal levels. While phase–amplitude coupling is most commonly observered in contemporary cognitive neuroscience (Lisman and Jensen 2013, Riddle et al. 2021), historical accounts of cross-frequency coupling focused on a special type of amplitude–amplitude coupling called harmonic resonance. In harmonic resonance, a standing wave possesses a finite number of resonant frequencies with numeric relationship to the original wave. Amplitude more readily spreads between frequencies with a harmonic relationship. For example, a recent study proposed that brain networks possess an intrinsic frequency of operation and the dynamic interaction of brain networks at neighboring frequency bands is governed by harmonic resonance (Atasoy et al. 2018). Recent work suggests that the phase–phase coupling can be estimated as a temporally stable integer relationship (empirically, an integer of 2) between the instantaneous frequency of signals from nested spatiotemporal scales (Klimesch 2013, 2018). Future research should investigate to what degree these different modes of cross-frequency coupling serve a similar neural function.
- Despite limited methods for recording ultra-fast protein dynamics (Smock and Gierasch 2009), there is evidence that proteins are observer windows. First, millions of atoms within a protein are synchronized into a single functional unit (Vattay et al. 2015; B. Zhang et al. 2017). Conformational changes on the scale of microseconds exhibit near-zero-phase lag (Bowman and Pande 2010, Chung et al. 2012). Second, proteins receive chemical and electrical signals from their neighbors, e.g. calmodulin (Frederick et al. 2007) and NMDA receptors (Mori and Mishina 1995). Communication via electrical activity in the movement of electrons and charged ions (B. Zhang et al. 2020) results in dynamic functional networks (Wei et al. 2016), called protein pathways, with complex systems of interdependencies that likely have rhythmic signatures (Smock and Gierasch 2009). Third, cellular-protein interactions in neurons could exhibit cross-frequency coupling. Calcium ion (Ca^2+^) concentrations fluctuate rhythmically at the cellular level (Woods et al. 1986) and are regulated by proteins on a microscopic scale, i.e. the Ca^2+^ wave (Rizzuto and Pozzan 2006). During action potential, a Ca^2+^ wave is generated in the intracellular space of neurons (Spruston et al. 1995, Smetters et al. 1999). This Ca^2+^ triggers proteins and protein pathways (Ghosh and Greenberg 1995, Berridge 1998). Thus, Ca^2+^ waves exhibit bidirectional influence across spatiotemporal scales (Cancela et al. 2002).
- The intrinsically subjective nature of consciousness may preclude the possibility of ever definitively distinguishing where consciousness resides. Nonetheless, evidence for or against multiple streams of consciousness might be gained by expanding investigations of existing lines of research, e.g. dissociative identity disorder (DID) and hypnosis. Cognitive load particularly compromises conscious processing (Sweller 2011), however, if consciousness is fundamentally divided then cognitive load given to one stream might not impact performance of another stream. This leads to the intriguing prediction that situations in which suggestive cases of parallel streams of consciousness have been reported might be clarified by investigating the capacity of an individual to simultaneously carryout tasks that normally would compromise one another. For example, if one personality of a DID patient is given the task of holding a digit set in mind, while the other is given a demanding task to complete, e.g. counting backwards, is the patient able to avoid the load costs typically observed in this context? A similar approach might be used in hypnosis by giving the so-called “hidden observer” a cognitive load task, while the hypnotized subject engages in another cognitively demanding task. Evidence of reduced impact of cognitive load in cases where parallel streams have been intimated would add weight to the argument that parallel streams of consciousness are possible, and would thus favor the emergent and panpsychic hierarchical consciousness models.
- If consciousness uniquely emerges at a certain level, then that threshold level should be accompanied by information integration processes that are not observed at lower levels. For example, if the apex is the unique home to consciousness, then its information integration processes should be qualitatively different from those observed at lower levels. Alternatively, if the emergent hierarchical consciousness view is correct, then information integration processes should be largely analogous between higher levels, but qualitatively different at lower levels. Finally, the panpsychic hierarchical consciousness view would predict generally analogous information integration processes across all levels of nested observer windows. Admittedly, we are not yet able to identify with sufficient specificity the information integration processes that could address the above conjectures, nevertheless they do not seem necessarily beyond reach. For example, IIT (Tononi et al. 2016) offers a sophisticated, if somewhat controversial, approach for quantifying the information integration processes that take place at assorted levels in the brain. Accordingly, if the ratio of phi (their measure of integrated information) between one level and the next remains largely constant between levels this would be an argument for a panpsychist view, whereas if the ratio changes markedly at a particular level this would be an argument for an emergentist or apex view. Critically, while this analysis is consistent with approaches taken by IIT, it does not depend on its assumptions. In principle, other characterizations of information integration/compression, e.g. (Fanelli 2019) or (Schmidhuber 2008), might be equally, or perhaps even more amenable to the task.

## The cognitive theater: binding of experience by synchrony

“[T]he way reality presents is not made up of lots of little sensations occurring in some stable space, not broken up into lots of little, individual sense [windows], but instead complete phenomena [are] perceived as consolidated in a more integrated way, meaning that they are formed together, with space, awareness, and all the different types of sense qualities happening all together to make up the objects in the sensate world, and even all of those objects in the world arise in these integrated wholes, consolidated swaths of moving space that contain all those things within them.” – Daniel M. Ingram (Ingram 2018)

A theater presents a narrative rendition of a series of events. These scenes can be in abstract form, telling a story through allegory, or faithfully detail the particulars of an event. Other renditions are unreliable in their message, e.g. told from the perspective of an antagonist. Like a play, observer windows in the NOW Model present a unique perspective on events through a specific lens. Furthermore, the actors, props, and immersive backdrop are orchestrated into a single narrative experience. In similar form, an observer window receives information from its neighbors, laterally via coherence or vertically via cross-frequency coupling, and this information is akin to elements of the play (actors, props, and backdrop) that are bound together into a single narrative stream within the observer window.

### Functional units

**Figure 5.**
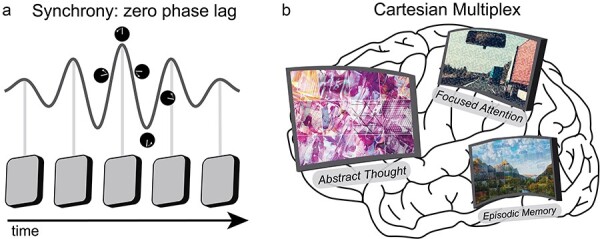
The Cognitive Theater and Cartesian Multiplex. (A) Observer windows are defined by synchrony, high internal correlation of its parts with zero-phase lag. The processing speed is represented by the precession of a clock in phase with each peak representing a refresh of the observer window. (B) As in the multiple drafts model by Daniel Dennett, multiple accounts of reality coexist within the brain. At any given time, a subset of observer windows (theaters) of various attentional emphasis (denoted by size) dominates subjective experience. Each theater in the Cartesian Multiplex possesses a different time signature, depicted here in the density of mosaic tiling. Slower percepts are typically more abstract (left; low-density tiling) and faster are more detailed (top; high-density tiling)

Observer windows are the functional units of the brain. It is often taken for granted that such unified structures exist. Here, we take care to specify the criteria for what can be defined as an observer window. We propose that the components within an observer window must exhibit zero-phase lag synchronization (Fig. 5a). Zero-phase lag means that the components are highly correlated, exhibit a matched peak frequency of activity, and essentially behave as a single whole unit. Using spectral decomposition of the electrical signal, an observer window can be identified via a Gaussian distribution of increased spectral power in a frequency band that is elevated above the background signal (He 2014, Voytek et al. 2015b, Donoghue et al. 2020). This identification method applies at the level of cortex with relatively slow processing speeds and at higher frequencies to identify neurons. Note that the spatiotemporal scale at which the measurement is acquired is critical as observer windows are band-limited to a particular spatial frequency and temporal frequency.

An observer window is created by the formation of a barrier from the environment. Synchrony in neurons is created by a phospholipid bilayer membrane that acts as an electrical capacitor and insulates neurons from the environment (Gentet et al. 2000). The resulting electric field within a neuron synchronizes electrical impulses that enter the membrane with nearly zero-phase lag (Hines and Carnevale 1997). The integration rate of a neural observer window is determined by the duration of voltage differentials generated by inputs, on the order of milliseconds (Hines and Carnevale 1997). Similarly, the architecture of brain nuclei in cortex utilizes synchrony to create observer windows. Within a subcortical nucleus or a region of the cerebral cortex, hundreds of thousands to millions of neurons are organized into cortical columns with parallel axons that enable ephaptic coupling (Bokil et al. 2001, Anastassiou et al. 2011, Buzsáki et al. 2012). Synchronous spiking activity and sub-threshold local-field potentials generate an electric field that is susceptible to mesoscale forces (Buzsaki 2006, Fröhlich and McCormick 2010, Agarwal et al. 2014) that modulates swaths of neurons within a nucleus at near zero-phase lag with a typical processing speed in the sub-second range (Tsodyks et al. 1996, Buzsáki 2002).

While observer windows are often instantiated in fixed anatomical structures, observer windows can also be instantiated as a stable meta-structure within a dynamical system, e.g. two neurons are fused into a single functional unit via a gap junction that synchronizes the electric fields of the neurons (Goodenough and Paul 2009). Note, infrastructure to achieve synchrony is required and cannot be stochastically achieved in the environment. Furthermore, the temporal extent and continuity through time of an observer window is enabled by fixed anatomy, e.g. neurons survive for a lifetime.

Evidence from cognitive neuroscience suggests that there is a profoundly rhythmic nature to the activity of cortex and neurons (Buzsaki 2006). This suggests that an observer window is rhythmically refreshed and information is transmitted outwards with a rhythmic signature. Nonetheless, observer windows likely maintain a predictive model that is stable through time and rhythmically updated by new information. In predictive coding, activity corresponds to deviations from prediction (Friston 2010, Arnal and Giraud 2012, Seth et al. 2012). Observer windows likely transmit information with a rhythmic signature and yet the experience from within is continuous (see VanRullen 2016 for a discussion). As will be discussed in “The apex observer window” section, the slow processing speed of the apex precludes an interpretation where each refresh of the observer window is a new “moment” in time, but likely represents increased likelihood for a change in the focus of attention.

### Binding by synchrony

In psychology, the binding problem is the question of how a single perceptual gestalt is generated from diverse information streams (Treisman 1996). The NOW Model is compatible with the *binding by synchrony* solution (Singer and Gray 1995, Engel and Singer 2001, Hunt and Schooler 2019) by which information is accumulated and bound into a single integrated representation by synchrony. The NOW Model posits that the peak frequency of synchrony in an observer window sets the processing speed at which new gestalt representations are generated. This processing speed may correspond to the phenomenal experience of time as each gestalt creates a sort of event boundary in the unfolding narrative (Clewett et al. 2019). Critically, information acquired in separate cycles is integrated into different gestalt representation (VanRullen and Koch 2003, VanRullen 2016). For example, visual information is chunked into discrete perceptual cycles around 10 Hz corresponding to electric fields in visual cortex (Callaway and Yeager 1960, VanRullen and Koch 2003, Hanslmayr et al. 2011, VanRullen 2016) and linguistic phonemes around 8 Hz in auditory cortex (Ghitza 2013, Peelle et al. 2013, Poeppel and Assaneo 2020). The integration rate of observer windows describes how some perceptions are fused and others flicker (Venables and Warwick-Evans 1967, Samaha and Postle 2015), and why attention seems to blink out between each object of attention (Raymond et al. 1992, Shapiro et al. 1997, Fiebelkorn and Kastner 2018, Helfrich et al. 2018).

Non-invasive brain stimulation methodologies such as rhythmic transcranial magnetic stimulation (Romei et al. 2016) and frequency-specific tACS (Riddle and Frohlich 2021) allow for the causal investigation of the role of oscillatory electrical activity in cognition. These studies demonstrate that not only are neural oscillations correlated with specific cognitive processes but brain stimulation techniques that specifically drive these neural oscillations modulate the associated cognition. For example, theta oscilaltions in lateral prefrontal cortex and alpha oscillations in posterior parietal cortex were associated with the prioritization and suppression of working memory representations, respectively (Wallis et al. 2015). When rhythmic transcranial magnetic stimulation was applied to these regions, the cognitive processes were facilitated by brain stimulation that aligned with endogenous oscillations and were disrupted by misaligned brain stimulation (Riddle et al. 2020a). This study, and others like it (Sauseng et al. 2009, Thut et al. 2011, Albouy et al. 2017), demonstrate that the rhythmic electrical activity of functional brain areas serves a causal role in cognition.

One consequence of a modular organization is that the speed of movement through time differs by observer window and the brain collectively comprises a variety of processing speeds. Furthermore, the same information is processed iteratively in many observer windows, e.g. at multiple stage of the visual processing pipeline (Herzog et al. 2016). When considering observer windows at different scales, the NOW Model suggests that the sequence by which information is integrated will be dictated by the flow of information through the nested hierarchy. In perception, slower observer windows will often lag in their experience relative to faster observer windows (Bartels and Zeki 1998), but this flow should be reversed during goal-directed behavior where the will to act first arises in slower observer windows.

The subjective experience of time dilation and time contraction might be accounted for in the NOW Model. When the apex observer window shifts attention towards slower or faster observer windows, phenomenal time changes. These fluctuations are gradual in typical experience, but change dramatically under extreme circumstances, such as the experience of life in slow motion during a traffic collision and the unexpected passage of an hour while day-dreaming on a nature walk. Recent experiments indicate that environmental conditions can profoundly impact the phenomenal experience of time. For example, one group showed that participants demonstrated a marked subjective experience of time dilation during 2–3 s of freefall (Stetson et al. 2007). However, findings are more varied in whether experientially induced changes in phenomenal time are associated with changes in temporal acuity. For example, Stetson and colleagues did not find an effect of free fall on temporal acuity for quickly flickering visual stimuli (Stetson et al. 2007), suggesting that threat brings faster observer windows into attention but does not accelerate the rate at which information is integrated in those observer windows. However, other studies found that the processing speed of visual observer windows shifted in frequency. For example Hagura and colleagues instructed participants to either press a button or actively perform a reaching movement while making a visual discrimination judgment (Hagura et al. 2012). They found that the act of reaching, but not button pressing, led to both increases in time dilation and enhanced temporal discrimination.

Additional studies demonstrated that experimental demands modulate temporal processing. For example, in one study, brain activity was recorded during a visual task that required participants to either integrate more information over time or to segregate information in time, and the investigators found that occipital alpha oscillations decreased or increased, respectively, to meet these task demands (Wutz et al. 2018). These more recent studies suggest that attention can modulate which observer window is dominant in processing the visual information, which in turn impacts both the experience of the passage of time and the acuity of temporal judgments. More research is required to unpack how attention impacts time perception and the conditions under which changes in the subjective passage of time do and do not reflect changes in temporal acuity.

### Cartesian multiplex

In the NOW Model, there is a multitude of theaters of experience; a view that we term the Cartesian Multiplex (Fig. 5b) in deference to Dennett’s (1991) critique of the Cartesian Theatre (Dennett 1991). To illustrate the Cartesian Multiplex, we provide an example of typical conscious experience:

*When driving, you start to mind-wander, pondering the nature of consciousness. Without warning, you suddenly realize you are almost at your destination. You wonder where the time went. Now your attention is focused on navigating which freeway exit to get off and on what side streets to turn. You are acutely aware of the lack of awareness paid to driving, but you do not notice that the wandering mind kept wandering while you were focused on navigating the side streets. When parking your car, you revisit the wandering mind. There is a fresh batch of theories and models that have materialized. You diligently proceed to review those new ideas as you absent-mindedly walk up the driveway*.

Not only does an observer window process the act of driving when you are focused on mind-wandering but also when you are focused on driving, an observer window keeps mind-wandering. This common situation highlights an outcome of the NOW Model: there coexists many autonomous models of the world within a single brain. Daniel Dennett described the Cartesian Theater as a single stage upon which various percepts unfold with the self as the observer (Dennett 1991). His description was used to illustrate the trouble with proposing a dualistic framework with observer and theater. In the NOW Model, we augment this metaphor to describe the brain as a nested Cartesian Multiplex, whereby the supervening apex observer window is aware of lower-order observer windows that populate its stage. Furthermore, only a subset of observer windows occupy the focus of attention and other cinemas continue their show without observation from the apex. Thus, the experience of a subjective vantage point at any given time is veridical; and yet, this vantage point will change to encapsulate drastically different perspectives at the exclusion of others.

The Cartesian Multiplex is most evident in a variety of extreme situations, which is indicative of its presence in typical consciousness. After callosotomy, split-brain patients demonstrate independent desires, beliefs, and goals in each cerebral hemisphere (Sperry 1984, Gazzaniga 1995, Volz and Gazzaniga 2017). With disrupted interhemispheric communication, the underlying Cartesian Multiplex is more apparent. For example, in one split-brain patient one hemisphere believed in God and the other did not (lecture by V.S. Ramachandran discussed by Platchias 2014). While experiments in split-brain patients reproducibly dissociated hemispheric specialization, these findings were met with controversy as the notion of a split mind is fundamentally unsettling to some people. Callosotomy either produces two apex observer windows at the level of the cerebral hemispheres, or more simply results in an accentuation of the modularity of observer windows at the level of cortex leaving the single apex observer window intact. Aspects of the induced modularity from complete callosotomy are present in the partial callosotomy, and even with complete callosotomy, indirected pathways through subcortical structures are intact (Gazzaniga 2005).

Inversely related to the split-brain case, the twins Krista and Tatiana Hogan are conjoined at the skull (craniopagus) with a white matter bundle that links each girl’s thalamus to the other (Cochrane 2021). The twins exhibit two different personalities and report unique experiences, but are able to hear the thoughts of the other twin and to be impacted by the perceptions of the other. However, this access must be intentionally acquired and is only occasionally accessed spontaneously (Cochrane 2021). Critically, the twins engage in dialogue with each other directly via neural signaling and must collectively decide on future actions to coordinate their conjoined bodies. The twins can relinquish or take control of certain limbs from the other. From the perspective of the NOW Model, the experiences of the Hogan twins are not quite as unusual as might be imagined. Internal debate at the level of cortex is typical of normal consciousness in the NOW Model and accentuated with the leap from two brain hemispheres to four. We speculate that the twins retain separate apexes and potentially exhibit lateral coherence at the apex level, which is not typical in human subjective experience. Alternatively, the twins may share a subset of their nested observer windows and only communicate at levels beneath the apexes. See (Cochrane 2021) for a counterargument that the girls may share a single consciousness or at least partially share consciousness.

Recent attention to peculiar animal models further illustrates the Cartesian Multiplex (Godfrey-Smith 2016). Each tentacle of an octopus appears to possess sufficient neural complexity to exhibit autonomous behavior and unique neural activity (D. B. Edelman et al. 2005). By encouraging independent processing within each tentacle, the octopus increases its dynamic flexibility to respond to the environment. At the same time, the octopus as a whole coordinates fluid movement and goal-directed behavior. This design is explainable in the NOW Model in that multiple observer windows coexist at the spatiotemporal level of each tentacle and are nested within an apex observer windows. However, this organization may differ from humans in that there may be reduced communication between the apex and the tentacles of the octopus relative to the equivalent structures in the human brain.

Other curious states of mind may also be explained by the Cartesian Multiplex. In hypnosis, the individual undergoing hypnosis will often describe the events of a memory from a third-person perspective where the person speaking is dissociated from the feelings of the event, known as the hidden observer phenomenon (Hilgard 2017). Furthermore, in the hypnotic state, these hidden observers access information unknown to the individual, often explained by access to information that is latently held in the unconscious depths of the brain (Császár et al. 2016). Alternatively, the NOW Model predicts a separation in knowledge base between observer windows each with a unique perspective on the whole organism. Hypnosis could be a means by which certain observer windows are provided with privileged access to others not typically afforded in normal consciousness.

Dissociative Identity Disorder (DID) is consistent with the ontological reality of the NOW Model. In the presence of extreme trauma, such as shame and attachment in early childhood abuse, DID can arise in which distinct “alters” coexist within a single individual (Dorahy et al. 2014). When the personality is fragmented in DID, a perfusion functional MRI analysis found distinct activity patterns corresponding to different alters; these MRI differences could not be simulated with trained actors (Schlumpf et al. 2014). Nonetheless, DID is considered controversial in the face of considerable evidence to its validity (Christensen 2022). We posit that this controversy might stem from an inability to reconcile DID with contemporary neurobiological theories of consciousness. In contrast, the NOW Model posits autonomous observer windows and a nested hierarchical organization. DID might arise from a breakdown in communication between observer windows and reduced top-down control from the apex (and potentially partial dissolution of the apex). Investigations of the dreams of those suffering from DID found that alters were represented as different dream characters each perceiving a single dream event from a unique perspective and role (Barrett 1994). This observation suggests that dreams may provide unique insight into the ontological reality of consciousness (Kastrup 2018). The dreamscape might be populated by observer windows taking the form of dream figures, and yet unlike waking consciousness, many observer windows express themselves creatively instead of faithfully representing the external environment. Finally, the recent increase (circa 2020–2022) in DID (Christensen 2022), while perhaps a social contagion, may represent a cultural recognition of hierarchical consciousness wherein DID is a spectrum disorder.

### Testable predictions and open questions

- While regions of subcortex display clear delineation, e.g. hippocampus and nucleus accumbens, the cerebral cortex has fuzzier boundaries between functional areas. The NOW Model suggests that functional areas will be identified by internal electrical synchrony (zero-phase lag) and weaker coupling, coherence (non-zero phase lag), with neighboring functional areas. Investigators should compare coupling strength within versus between retinotopic maps in primary, secondary, and tertiary visual cortex (Silver and Kastner 2009).
- Other cells, such as glial cell, or nuclei in the peripheral nervous system, might also act as observer windows. Furthermore, observer windows may exist at spatiotemporal scales relevant to cognition at more foundational level, e.g. the cytoskeleton (Pinotsis et al. 2023), the molecular scale (Fisher 2015), or at supervening levels, e.g. whole-brain structure (Pang et al. 2023), the electric field of the body (Klimesch 2013, 2018), or between individuals in social settings (Gallagher and Frith 2003, Dikker et al. 2017, Parkinson et al. 2018).
- Observer windows exhibit discrete spatial and temporal constraints, so an open question is the regularity at which observer windows are found within the hierarchy. In fractal mathematics, the Hurst exponent describes the ratio at which a function is invariant after a transformation (Bunde and Havlin 1994), i.e. upon a determinable amount of zooming, the initial pattern repeats. With neither an arbitrary, nor infinite, number of spatiotemporal scales at which observer windows are found, mathematical principles may determine the spatiotemporal separation between observer windows of neighboring levels.
- The NOW Model suggests that rhythmic activity in observer windows is the basis for quantifying processing speed. Future work should continue to explore the time constants, and potential discretization, of cognitive processes. Some domains of psychology established spatial correlates such as the precuneus for mind-wandering (Christoff et al. 2009), but currently lack a rigorously defined temporal signature. Critical, yet unknown, parameters for this model are the time constants for which an observer window is opened and closed. We suggest that the cycle characteristics of electrical oscillations, e.g. peak-trough asymmetry (Cole and Voytek 2019), are potential physiological correlates of open-close time constants.
- While some evidence exists for a time lag between nested scales (Zeki 2003) and the extended duration of higher-order observer windows (Hagura et al. 2012), further research is required to substantiate these claims. For example, abstract reasoning and metacognitive reflection should shift higher-order observer windows towards a lower frequency with a corresponding increased speed in the subjective passage of time.

## Society of mind: dialogue through coherence

With many cognitive theaters, the brain exhibits a rich dialogue between observer windows. Like two people in conversation, the NOW Model proposes that when observer windows communicate information is transferred between them with a conduction delay determined by physical limitations. Consistent with this proposal, the brain is theorized to be a modular network of isolated cognitive systems that transiently interact (Fodor 1983, Gazzaniga 2000, Zeki 2003). In a modular system, the organism gains cognitive flexibility by encouraging conflicting perceptual and action schemas to develop, and at the same time, modules must cooperatively work towards higher order goals. To this end, observer windows initiate dialogue and gather information from neighbors.

### Coherence as dialogue

**Figure 6.**
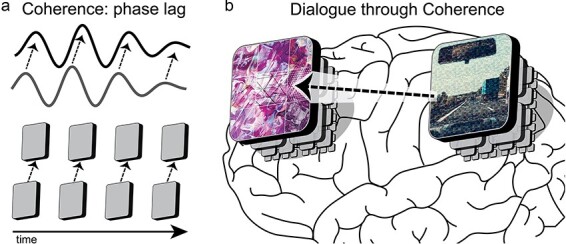
Society of mind via dialogue. (A) In the NOW Model, coherence is a weak form of coupling that transiently aligns two observer windows and facilitates the sharing of information. With a profoundly modular organization, the multitude of cognitive theaters in the brain engage in a dialogue to achieve shared goals. (B) For example, an observer window in prefrontal cortex and another in posterior parietal cortex, each comprising nested subwindows, are engaged in dialogue through coherence. The prefrontal observer window is depicted as processing abstract thought, while the parietal observer window is focused on driving a car. These observer windows will transiently engage in dialogue to share pertinent information. In this example, a single arrow from parietal cortex to prefrontal cortex might correspond to an interrupt signal as an event on the highway (processed in parietal cortex) requires another perspective (from prefrontal cortex)

Coherence is similar to synchrony except that the communication is fundamentally delayed—there is a non-zero phase lag between the regions as they become transiently aligned in time (Fries 2005, 2015, Von Stein and Sarnthein 2000; X.-J. Wang 2010) (Fig. 6a). A metric that captures this form of weak coupling is weighted phase lag index (wPLI) that quantifies a systematic non-zero phase lag between two independent signals (Vinck et al. 2011). Spatially separated regions of cortex transiently align their electric field oscillations such that each neural population will be depolarized and hyperpolarized with a consistent phase lag to optimize synaptic transmission; i.e. “neuronal communication through neuronal coherence” (Fries et al. 2001, Fries 2005, 2015). Here, we adopt this terminology from Pascal Fries with only minor alteration to emphasize the autonomy of each observer window: “dialogue through coherence” (Fig. 6b). At a given time, only a subset of neural pathways are relevant to the current context (Serences and Yantis 2006). Coherence allows for increased flexibility in a dynamic world because a weak synaptic pathway can be temporarily strengthened by coherent electrical activity (Bassett and Sporns 2017). For example, the perceptual systems relevant to the formation of a new memory exhibit theta-frequency (4–8 Hz) coherence with the hippocampus in order to laterally share information with hippocampus to be bound into an episodic memory (Schott et al. 2013, Backus et al. 2016, Clouter et al. 2017), but these regions fall out of coherence soon after encoding is completed.

Behavioral systems facilitate interregional coherence via external driving forces. For example, the speech envelope of linguistic articulation exhibits a pronounced peak in the theta-frequency (Ghitza 2013, Park et al. 2015) as does the saccade rate in visual exploration (K. L. Hoffman et al. 2013). When linguistic articulation is received by another person, their auditory cortex entrains to the waveform of the speech envelope. Similarly, the movement of the eyes entrains the visual cortex to the rhythm of the movement. With multiple behavioral systems entraining perceptual observer windows to the same rhythm, these disparate regions more readily share information. For example, presentation of flickering video and audio stimuli in-phase in the theta-frequency was more accurately remembered than misaligned stimuli or stimuli in a different frequency band (Clouter et al. 2017). This study also demonstrated transient coherence of theta oscillations in visual and auditory cortex during the presentation of these stimuli (Clouter et al. 2017).

In addition to behavioral entrainment, the NOW Model posits that observer window possess the capacity to initiate dialogue with their neighbors. Dialogue could be initiated by sending a volley of entraining signals to a target observer window. The message is received when transient entrainment is observed in the receiver. When initiation is successful, the observer windows may enter into a sustained state of coherence for dialogue. During a visual search task, for trials in which the target “popped out” from a search grid, a region in parietal cortex directed activity in the gamma-frequency band (30–60 Hz) from visual processing areas to motor preparation areas, but when the target was hidden among distractors, a region in frontal cortex directed activity in the beta-frequency band (15–30 Hz) from motor preparation areas to visual processing areas (Buschman and Miller 2007). This experiment demonstrated that different contexts required different regions to send or receive information. We propose in the NOW Model that observer windows rapidly initiate dialogue and aquire information from each other. In the study above, these complex dyanmics all occurred within 50–300 ms after presentation of the search grid. This time scale corresponds with the processing speeds of cortical observer windows. Thus, cortical observer windows often converse quickly and the apex observer window becomes privy to these conversations only after their occurrence, if at all.

Beyond correlational evidence, studies delivered brain stimulation in humans and demonstrated that coherence between brain regions facilitates the sharing of information and contributes to cognitive function. For example, theta-frequency tACS delivered in-phase to both frontal and parietal cortex was repeatedly demonstrated to improve cognitive control (Polanía et al. 2012, Jaušovec and Jaušovec 2014, Violante et al. 2017). When recording invasively from the human brain, in-phase direct electrical stimulation to frontal and parietal cortex during a working memory task increased non-zero phase coherence between the targets and improved performance (Alagapan et al. 2019). Thus, causal evidence from brain stimulation studies confirmed that observed coherence during cognition is causally related to cognitive function.

### Corporation of cognitive theaters

Beneath the apex of the nested observer windows hierarchy, an underlying corporation of semiautonomous individuals are encouraged to maintain conflicting beliefs and goals. Observer windows regularly engage in a debate between mutually exclusive worldviews. We hypothesize that during periods of increased conflict coherence will increase between regions of the brain corresponding to dialogue between competitive observer windows. Furthermore, particular observer windows likely specialize in encouraging coherence between those with conflicting percepts and action plans. For example, experimentally induced cognitive dissonance reveals activation in dorsal anterior cingulate cortex to monitor conflict (Van Veen et al. 2009), and certain regions are known to initiate coherence between other regions (Saalmann et al. 2012).

Cognitive dissonance in psychiatric illness is when conflicting beliefs are simultaneously maintained even though they are fundamentally incompatible (Harmon-Jones and Mills 2019). In the NOW Model, cognitive dissonance arises from unresolved disagreement and antagonism between observer windows. Cognitive dissonance is by definition not recognized, and thus, dialogue through coherence is not present between the observer windows with conflicting views. Some psychiatric symptoms, such as auditory hallucinations, manifest as reduced coherence from an executive control observer window in prefrontal cortex to an auditory observer window (Lawrie et al. 2002, Vercammen et al. 2010). This situation might be understood as a failed cooperation between a goal-oriented observer window and an imaginative perceptual observer window. Dissociative disorders such as auditory hallucinations are thus conceptualized as cognitive dissonance under the NOW Model, where cognition is recognized as co-occuring in both regions as opposed to traditional views that cognition only occurs at the macroscopic scale. This shift in framing for psychiatry is perhaps best exemplified by internal family systems psychotherapy, in which the mind is conceptualized as a family of personalities with independent desires and roles (Schwartz and Sweezy 2019). Internal family systems psychotherapy is conducted by acknowledging competing personalities within the individual and developing tools for creating harmonious dialogue between internal family members. In the NOW Model, coherence between observer windows reflects productive conversation between members of the internal family and this productive dialogue can be cultivated through the development of internal conflict mediation strategies. For example, removing self-judgment for aspects of the self that are viewed as underdesirable and instead recognizing the function and perspective of alienated members of the internal family.

Recognition of hierarchical consciousness is therapeutic within the internal family systems context, and yet this conceptualization of the self invokes cultural associations with DID. The recent uprise in DID illustrates the ease at which people can recognize the reality of a Cartesian Multiplex in the brain and yet the cognitive dissonance likely stems from psychological trauma and is not inherent to acknowledging hierarchical consciousness (Christensen 2022). The pheneomonology of DID is highly consistent with how the NOW Model describes the level of cortical observer windows. For example, alters in a person with DID exhibit nuanced relationships whereby one alter is aware of some but not all alters. Indeed, if alters in DID are different observer windows, then knowledge transfer between alters would occur via dialogue through coherence. This transfer is transient by nature and only provides a limited understanding of another observer window. Furthermore, the internal family systems framework and DID identifies specific alters as serving privileged roles as conflict meditators. These conflict mediators might be localized to connector hubs in the brain, regions with a high degree of connectivity to the rest of the brain (Bertolero et al. 2018). Once again, DID is consistent with scientific models of the brain’s modular organization. The principle of coherence in the NOW Model provides a mechanistic framework for testing the empirical validity of internal family systems and may come to bear on cognitive dissonance in dissociative disorders.

### Testable prediction and open questions

- Researchers often conflate synchrony and coherence. If two signals are coherent, then the signals will also be correlated. However, two correlated signals will not necessarily exhibit non-zero phase lag. For example, the use of correlation for connectivity analyses in functional MRI does not suggest zero-phase lag between regions of cortex, but instead reflects the limited temporal resolution of functional MRI. While a correlation implies synchrony, disparate regions of cortex are likely not in synchrony, but are rather coherent, as evidenced by magnetoencephalography data with sufficient temporal resolution (Clouter et al. 2017). For analogy, the ability to run a Fourier transform does not in principle suggest the presence of oscillatory activity (Donoghue et al. 2020).
- The NOW Model postulates that some observer windows act as moderators that coordinate dialogue between regions. Mechanistically, some nuclei in subcortex might specialize in driving coherence; e.g. pulvinar nucleus of the thalamus (Saalmann et al. 2012) or the claustrum (Vidyasagar and Levichkina 2019; Q. Wang et al. 2017).
- Investigations into whole-brain network properties find a critical balance between sparsity and density (Bassett and Sporns 2017). Whole-brain analysis in functional MRI uses network properties to quantify modularity (Bertolero et al. 2015, Gallen and D’Esposito 2019); however, this previous work conflates synchrony and coherence. Future research should first rigorously define synchronous units, i.e. observer windows. Then, patterns of coherence between observer windows could be characterized via existing modularity metrics wherein communication cliques might be formed with high within-clique connectivity and low between-clique connectivity. These analyses might give insight into the structure of dialogue between corporations of observer windows.
- The process by which observer windows initiate and sustain coherence should be further investigated. We hypothesize that brief rhythmic signal volleys create transient periods of coherence that might result in longer periods of sustained coherence. Furthermore, with sustained dialogue we might expect to see shifts in phase lag wherein one observer window leads the other and then roles are reversed.

## The apex observer window

A central question that the NOW Model attempts to address is the character and role of consciousness in the brain. Neuroscience continues to struggle to define a neural correlate of consciousness that produces a single locus of experience (Koch et al. 2016). Based on this persistent challenge in neuroscience, it is tempting to remove the requirement for a locus and to propose that the brain generates a multiplicity of cognitive theaters with the assertion that the self is an illusion, e.g. consequent from memory reconstruction. Indeed, one could utilize the NOW Model without the inclusion of an apex observer window and the resulting model would resemble a nested, multi-layered version of Dennett’s multiple drafts model (Dennett 1991). However, it is our opinion that the unitary subjective vantage point necessitates its inclusion in the NOW Model. The features thus far described generate specific predictions as to where future work should look to find the apex of the NOW hierarchy.

### The apex is slow

A key feature of the NOW Model is that observer windows exhibit a processing speed proportional to their size and scale. The apex observer window must be larger than the level corresponding to regions of cortex, and thus should be an order of magnitude slower. With cortical regions exhibiting synchronized electrical activity in the 1–50 Hz range, the apex likely resides in the “slow” frequency range from 0.1 to 1 Hz, which corresponds to 1 cycle per 1 s to 1 cycle per 10 s. As such, the apex observer window provides a cognitive theater with a temporal extension long enough to integrate the many different time signatures at the cortical level and to integrate across many cycles of each cortical rhythm. For example, when language is articulated in a speech envelop fluctuating at 8 Hz, entire sentences can be integrated into a single gestalt within a single slow cycle. Eight phonemes 125 ms apart become integrated into a 1-s sentence. Similarly, saccadic eye movements, which occur at ∼5 Hz, each provide a snapshot from the fovea of the retina to the visual cortex. While standing on a beautiful mountain vista, each of these saccadic snapshots are integrated into a comprehensive view of the landscape at the slow scale: five snapshots by the fovea 200 ms apart become a landscape at one second. Audio-visual integration at the scale of entire sentences and visual scenes is performed in the apex observer window at a slow speed. Goal-states that were found to be processed in the delta range (1–4 Hz), discussed in a previous section, are embedded within this apex level and gain access to an expanded capacity for contextual understanding built from slow multimodal sensory integration.

The slow timescale was proposed to be essential for consciousness by multiple researchers. For example, Biyu He and Marcus Raichle proposed that the slow-cortical potential (SCP) corresponds with conscious perceptual awareness as well as volition with the unique capacity to integrate information over long temporal windows and across a vast neural extension (He and Raichle 2009). The SCP takes from 1 to 10 s to unfold and in some studies was found to be correlated with the canonical response profile of the blood-oxygenation level-dependent (BOLD) signal from functional MRI (He et al. 2008, Khader et al. 2008). In a similar fashion, Georg Northoff noticed a paradoxical finding that slow and infraslow signals increase when consciousness is loss and yet when these signals are measured in the awake state they positively correlate with conscious processing (Northoff 2017). To explain this paradox, Northoff proposes that the key factor for consciousness is cross-frequency coupling between the slow apex scale and the faster cortical scale, referred to as “temporo-spatial nestedness” (Northoff and Huang 2017). These authors explore how anesthesia, deep-sleep, and seizure states, which are associated with a lack of consciousness, disrupt cross-frequency coupling between the slow scale and the level of cortex even though there is a pronounced increase in the amplitude of slow activity (He and Raichle 2009, He et al. 2008; J. Zhang et al. 2018), but see evidence that cross-frequency coupling is partially spared (Vanhatalo et al. 2004, Watson 2018, Liu et al. 2021). Thus, in their temporospatial theory of consciousness, Northoff and Huang concluded that the slow level is necessary for consciousness and that normal consciousness requires cross-frequency coupling between the apex level and the level of cortex (Northoff and Huang 2017). Similarly, Wolfgang Klimesch’s binary hierarchy brain body oscillation theory suggests that loss of body awareness in sleep and anesthesia corresponds to disrupted phase–phase coupling between the brain and body, and in particular uncoupling from the heart (Klimesch 2018, 2023).

The NOW Model asserts the importance of scale-free properties (He 2014) and cross-frequency coupling (Northoff and Huang 2017) as the primary means by which the apex observer window is connected to the observer windows in the cortex. However, there are a couple elements by which the NOW Model differs. First, Northoff’s temporospatial theory of consciousness proposes that slow activity is a neural predisposition for consciousness, but the level of cortex is the strongest neural correlate of consciousness (Northoff 2017). In the NOW Model, the apex of the hierarchy is viewed as an autonomous level that interacts meaningfully with semiautonomous lower levels. Observer windows beneath the apex contribute to, but are not synonymous with, human consciousness. As mentioned previously, the cognitive theater of the apex is populated by the contents of faster observer windows. Thus, the contents of our mind are faster than the slow scale leading to a misattribution of consciousness to the cortical level.

If the slow scale is the closest neural correlates to human consciousness, then one might wonder why this electrophysiological signal is not widely studied in neurocience. One reason is that the slow range is outside of the canonical range of electrophysiology such that many EEG devices are built with hardware limitations that automatically filter slow and infraslow activity (Monto et al. 2008). However, slow activity might be indirectly measured with fMRI as some evidence suggests that the BOLD signal tracks closely with the SCP (He et al. 2008). Thus, there might be a more fundamental difference between common human neuroscience methodologies such that fMRI systematically studies the apex level, whereas EEG is focused on the cortical level, but see Monto et al. (2008) for an example of a modified EEG system that found that the SCP was related to behavior. Furthermore, it may be difficult to distinguish between the activity of cortical observer windows and the apex when there is significant cross-talk between these systems. From what limited evidence is available, the processing speed of the apex observer window is most likely in the slow range, perhaps around 0.2 Hz which correspond to a 5 s cycle. This time constant aligns with the SCP in event-related analyses of EEG (Monto et al. 2008) and of BOLD signal fluctuations in fMRI (Duff et al. 2008). Another possibility is that the processing speed of the apex is around 0.02 Hz corresponding to a 50 s cycle based on a prominent peak around 0.02 Hz in EEG and fMRI (Watson 2018), but this speed appears to be too slow to account for the introspective time constants of subjective experience and we could not identify any published manuscripts advocating for this association.

Structurally, the apex of the NOW Model might be implemented via layer one interneurons (He and Raichle 2009). The “crowning mystery” refers to the mystery of what layer 1 of the cerebral cortex provides to brain function since it is sparsely populated by neuron bodies and displays widespread cortico–cortical connectivity (Ibrahim et al. 2020). Layer 1 might provide critical architecture for nesting an apex observer window on the sprawling cerebral cortex which would uniquely account for its functional role. With such a broadly distributed system, traditional experimentation had difficulty adequately characterizing the apex, e.g. focus on fast timescales, unimodal experiments that do not require the apex, and insufficient coverage in recording. Thus, there is evidence to suggest that studies focused on the slow timescale and the layer 1 cerebral matrix might find evidence for the apex observer window.

### Meta-awareness and meditation

It may appear strange to identify consciousness with such a slow processing speed, but the illusion of a faster conscious experience arises from the fact that our attention is always focused on the contents of our thought. Through mosaic tiling, the apex is rich with detailed images (generated by an observer window in visual cortex), high-resolution audio signals (auditory cortex), and nuanced abstract thought (prefrontal cortex). The layer of cortical observer windows nested beneath the apex provide content with a high degree of transparency. Their content is profoundly in our experience and our ability to understand fast and detailed stimuli leads us to assume that we are processing information at this faster timescale. In short, we mistake the content of mind for the mind itself. We are observers of this information, but we do not actively generate this content. What then is the content that we actively generate? We colloquially call the content we generate “thinking.” We hear some new idea, and we say to ourselves “hold on, let me think about that.” Seconds pass by as we gather our thoughts. We decide what to eat for dinner, choose the route to drive to work, write an essay, speak to our neighbor. These tasks, which are commonplace in our daily life, are within the slow timescale of multiple seconds. Thus, upon further reflection and by distinguishing apex consciousness from the contents within mind, this slower timescale can become more intuitively appreciated. To better understand how we could systematically mistake the contents of the mind for the mind itself, we must properly define how we reach introspective awareness.

**Figure 7.**
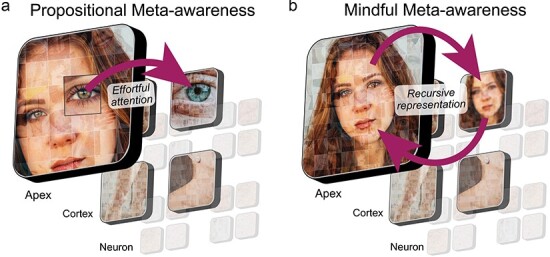
Two types of meta-awareness in the NOW Model. (A) Propositional meta-awareness is self awareness of some cognitive process. Effortful attention is required to sustain propositional meta-awareness and corresponds to increased top-down signaling from the apex to its nested subwindow generating the cognitive content. (B) Mindful meta-awareness is introspection on the self. Without the ability to witness itself directly, the apex must request and access a representation of itself within a nested observer window. This representation becomes recursive through prolonged interaction and is the subject of meditative practice

When queried about our mental state, the act of introspection generates a state of meta-awareness (J. W. Schooler 2002). At other times, we suddenly become meta-aware, e.g. you catch yourself mind-wandering when you intended to stay focused while reading (J. W. Schooler et al. 2011b). We distinguish two types of meta-awareness, which we refer to as propositional meta-awareness and mindful meta-awareness. The former is awareness of some cognitive process that is occurring in a nested observer window, and the latter is awareness of the apex observer window itself. For both types, meta-awareness is achieved through the interaction between apex consciousness and the observer windows nested within the apex. In the natural state, we are not self-aware of how attentional resource are allocated, nor of the diversity of cognitive processes intermingling within our mind. The contents of thought are seamlessly integrated into a gestalt such that the individual sources of information are obfuscated. Despite this seamless integration, we can become meta-aware of the operations of a nested observer window by focusing our attention on some specific content of information in our experience. Through the focusing of our attention on this internal cognitive process, the representation for that process is emphasized in the apex (Fig. 7a). Phenomenologically, propositional meta-awareness such as this requires sustained conscious effort to be maintained. The mechanism for meta-awareness is increased top-down attention from the apex observer windows to a nested observer window.

Propositional meta-awareness can be disruptive, e.g. overthinking what the body is doing in sports (Flegal and Anderson 2008). In the NOW Model, propositional meta-awareness generates top-down control signals that impact the ongoing processing within lower-order observer window by projecting abstract information into its cognitive theater. This information content can be disruptive to ongoing processes by changing the focus of attention of the subwindow or obfuscating its information content with abstract forms. While meta-awareness is sometimes disruptive, it may simultaneously enable improved capacity for creative higher-order thought. The act of propositional meta-awareness might be a unique mechanism by which higher-order representations are transmitted into subwindows. In contrast, a flow-state might be characterized in the NOW Model by a lack of propositional meta-awareness (Csikszentmihalyi 2014). Thus, a flow state would coincide with a marked reduction in top-down cross-frequency coupling such that subwindows are uninterrupted in their processing.

The second type is mindful meta-awareness, which is our ability to introspect upon the apex from the vantage point of the apex. In the NOW Model, the apex is fundamentally unable to directly introspect on itself because it is by definition an observation on the contents of its cognitive theater. Therefore, mindful meta-awareness requires a representation to be created within a nested observer window that provides this representation back to the apex (Fig. 7b). Most observer windows are focused on the content arising from its subwindows or transmitted from neighbors. However, when a lower-order observer window directs its lens of attention towards the macroscopic, then a representation of the higher-order observer window is generated. When the higher-order observer window focuses attention on the lower-order observer window that is focused back on it, then mindful meta-awareness is created through recursive representation. Thus, we are able to introspect on our own experience through the representation of our self in the content of our subwindows. As can be seen, meta-awareness is not direct and is subject to the cognitive biases of subwindow.

Meditation is often associated with increased propositional and mindful meta-awareness (Dunne et al. 2019). The NOW Model provides a compelling description of the nature of meditation. As an apex observer window, your relationship to the many observer windows at the cortical level will be complicated. We often find ourselves reacting to stimuli and sustaining emotions that are unwanted. These feelings might not arise directly within the apex but are generated by an autonomous observer window outside of our control. In meditation, we attempt to build a more robust relationship between our self and our subwindows. Meditation could be characterized as the creation of more sustained periods of meta-awareness between the apex and its nested observer windows. When first introduced to meditation, it can be frustrating to be thrown into the milieu of subwindows and to realize your lack of control. With time, meditators will increase their ability to exert control over their own mind, which could be operationalized as increased top-down control over subwindows in the NOW Model.

Although meta-awareness typically fosters the perception of unified consciousness, with practice sustaining meta-awareness (see Dunne et al. 2019) individuals may come to recognize the more fractured nature of consciousness. By sustaining propositional meta-awareness with many subwindows, subjective experience may broaden to accommodate the perception of these multiple subwindows as fractured from the self. In this manner, meditation may lead to the rejection of an internal narrative stream as the authoritative reflection of a singular perspective (Bernstein et al. 2019) and may (in some cases) further foster the view that the self itself is an illusion (Metzinger 2009).

### Testable predictions regarding meta-awareness

- Theoretically, it should be possible to negate conscious experience through electrical stimulation or some other manipulation that disrupts synchrony at the apex. This should result in a form of high-level anesthesia that spares the functioning of brain regions. This might be akin to sleep-walking and behavior would be primarily stimulus-response oriented.
- A single hemisphere can undergo sleep in some animals which suggests that there may be dramatic flexibility in what is encapsulated within the apex. It should be investigated the degree to which unitary consciousness possess a fixed anatomy versus flexibility to move among available lower-order observer windows.
- The presence of slow activity during anesthesia, deep sleep, and seizure suggests that consciousness is present during this time, but the reduction in cross-frequency coupling also observed means that the apex does not receive information from the cortex. Experience during this time likely comprises internally generated abstract content divorced from the external world. Future work could investigate whether cross-frequency coupling predicts depth of anesthesia.
- The information processed by the apex is proposed to encompass multiple modalities within a single theater of experience; however, there may be certain types of information that more readily enter the apex. For example, language is an effective means of capturing and expressing abstract information. Future research could investigate what types of information or cognitive tasks maximally engage the low-frequency activity of the apex.
- Research on advanced meditators could investigate differences in cross-frequency coupling between sustained propositional meta-awareness and mindful meta-awareness.

## Contemporary theories of consciousness

While the NOW Model includes a number of testable empirical assumptions of its own, its broad construal enables it to potentially accommodate (with alternative assumptions), and possibly even integrate, elements of a host of extant theories of consciousness such as higher-order thought models (G. M. Edelman and Gally 2013, Lau and Rosenthal 2011; D. Rosenthal 1993), global workspace (Mashour et al. 2020), and integrated information theory (Tononi et al. 2016). In this section, we highlight three theories of consciousness that each rely on one of the principles of the NOW Model or its associated metaphor. Higher-order thought theory relies on recursive scaling (D. Rosenthal 1993) implemented through reentrant circuitry (Lau and Rosenthal 2011), a putative mechanism for cross-frequency coupling in the NOW Model. Global neuronal workspace relies on synchrony (Mashour et al. 2020), which is the formation of an observer window in the NOW Model. Integrated information theory relies on connectivity (Tononi et al. 2016), which is coherence in the NOW Model.

**Figure 8.**
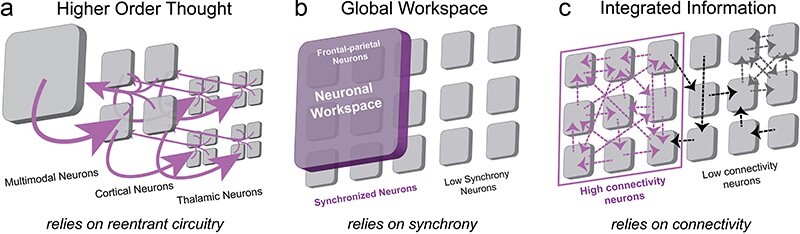
Principles of the NOW Model in theories of consciousness. The NOW Model provides a framework for comparing theories of consciousness. Purple denotes the primary mechanism by which consciousness is characterized in each theory. (A) The HOT theory describes consciousness as the awareness of mental states. The HOT theory places consciousness primarily in the self-reflection between first-order mental states and higher-order thoughts (purple arrows). Reentrant circuitry is proposed to provide a mechanism for HOT. The concept of mosaic tiling is akin to HOT. (B) Global workspace theory describes the emergence of a single “workspace” as consciousness. Global neuronal workspace posits that highly synchronized neurons create a single theater of consciousness akin to an observer window formed by synchrony. (C) The IIT is an analysis of the interconnectedness of neurons (dashed arrows) with a complexity value, phi, that roughly scales with the degree of connectivity. Consciousness in IIT is akin to a highly coherent network of neurons that create a dynamic observer window. In light grey, a smaller cluster of coherent neurons is depicted that does not create a conscious experience as described by IIT

A theory that exemplifies the first principle of the NOW Model is Higher Order Thought (HOT) theory. HOT theory asserts that in order to be consciously aware of a mental state there must be a higher-order thought which has access to that mental state (D. M. Rosenthal 1991). Thus, consciousness requires self-reference, similar to others that suggested recursion as a potential mechanism for consciousness (Hofstadter 2007). HOT theory further asserts that we are not typically conscious of the higher-order thoughts themselves, and thus, the NOW Model accounts for HOT theory in the principle of self-referential scaling (D. Rosenthal 1993), mosaic tiling (Fig. 8a). The original description of HOT relies on the metaphor used in the NOW Model for mosaic tiling, but does not make a statement regarding the mechanism. Recent extrapolations posit that the neural mechanism for HOT theory is reentrant signals through loops, e.g. the thalamocortical system (Lau and Rosenthal 2011).

Reentrant signaling theory utilizes the evolutionary architecture of the brain wherein newer structures were built on top of older structures, e.g. cerebral cortex on cingulate cortex on subcortex on thalamus on brainstem. Recurrent circuitry bridges these structures in loops where the same information re-enters the region of origin after processing elsewhere. Cognitive neuroscientists adopted HOT theory to explain the phenomenal experience of re-entry (G. M. Edelman and Gally 2013). While there are many forms of HOT theory in cognitive neuroscience, see Lau and Rosenthal (2011) for a summary, many of these theories assert that the prefrontal cortex provides the essential function of metacognition which is HOT’s key feature of consciousness. Reentrant signaling naturally gives rise to cross-frequency coupling such that the network-scale activity generates low-frequency rhythms that constrain local high-frequency afferent signals. While the metaphor of self-reference is consistent, the NOW Model posits that vertical cross-frequency coupling across spatiotemporal scales enables HOT, which is distinct from lateral connectivity in traditional HOT theory.

The global workspace model posits that consciousness exclusively arises at the scale of the largest and slowest observer window in the brain (Fig. 8b). This macro-scale observer window is created via synchrony and corresponds to the “global workspace” of global workspace models, e.g. (Dehaene et al. 1998, Baars 2005, Baars and Franklin 2007), within which information across modules of neurons is integrated into a singular information stream (Baars 1997, 2005). The global workspace could correspond to a tightly synchronized network of prefrontal and parietal neurons (Mashour et al. 2020), a single multi-modal association nucleus at the apex of the prefrontal hierarchy, e.g. anterior middle frontal gyrus (Nee and D’Esposito 2016), or it could be distributed across the brain (Freeman 2015). The global neuronal workspace model relies on the principle of synchrony and on the metaphor of a single theater, as in the NOW Model, for an explanation of how consciousness emerges. Of note, the global neuronal workspace model only considers biological activity at a particular spatiotemporal scale—that of the neuron, whereas the NOW Model posits there is behaviorally relevant information processed at many spatiotemporal scales and the apex observer window functions as a global workspace. It is unclear how the global workspace model resolves the emergence of a single consciousness from one set of synchronized neurons versus another.

While global workspace theories emphasize a synchronized observer window that is the location of consciousness, an alternative theory is the integrated information theory (IIT) which posits that the density and complexity of connections between neurons is critical to the formation of consciousness. Here the second principle of the NOW Model, coherence, is the critical metric of interest because this is the foundation for connectivity between two observer windows (Fig. 8c). In IIT, information is defined as the degree to which the current brain state predicts the next brain state and the level of consciousness in a region of the brain, i.e. PHI (Tononi et al. 2016). This is approximated by the bisectional cut that can be drawn through its network of interconnected neurons which maximally reduces the accuracy of this prediction, i.e. the minimal information partition (Toker and Sommer 2016). Thus, consciousness in IIT is not defined by the emergence of an observer window via synchrony, but instead every network of interconnected neurons will possess some degree of consciousness that scales with the degree of interconnectedness. IIT is consistent with the mechanism of coherence but claims not to support the metaphor of a society of mind as in the NOW Model. Instead, IIT asserts the exclusion principle whereby the network with the greatest PHI becomes the substrate for consciousness at the exclusion of all other networks. IIT is typically defined with neurons as the units between which connectivity plays a role; however, to apply IIT to other spatiotemporal scales requires a definition of the units. One strength of the NOW Model is that it clearly delineates the definition of an observer window through synchrony and the interaction between observer windows through coherence. The NOW Model provides a simple means of contrast between the global neuronal workspace and IIT as the first relies on synchrony and the latter on coherence.

We selected these theories because they highlight specific aspects of the NOW Model. Not only do the principles of the NOW Model come to bear on each theory, but the metaphors at their core are largely consistent. While the researchers behind these three theories often characterize their models as in conflict with one another, the NOW Model might resolve much of the perceived conflict by suggesting that each theory emphasizes different aspects of the biological mechanisms underlying consciousness, and each of these principles of the NOW Model are essential to conscious experience.

## Conclusions

The NOW Model proposes that consciousness is positioned at the apex of a hierarchical system spanning the scale of cortical regions to neurons and potentially to subcellular structures. Our model utilizes three core metaphors to describe hierarchical consciousness where each metaphor is quantified by a unique neural signature. First, observer windows are nested within a hierarchy such that each observer window integrates a mosaic representation of the information in its subwindows. Through mosaic tiling, perceptual information builds in abstraction when moving up the hierarchy and abstract action plans are disseminated when moving down the hierarchy. Second, observer windows generate a cognitive stream, like a theater, that integrates information with a unique narration. Third, a dialogue unfolds between neighboring observer windows to resolve conflicting perceptions and action plans. Altogether, the NOW Model captures a wide range of subjective phenomenology and generates testable predictions for experimental research.

The most unique motif of the NOW Model is that the observer windows are nested within each other across spatiotemporal scales. The theater of cognition within each observer window is akin to a mosaic tiling where each tile is itself a mosaic tiling. Mechanistically, a mosaic tiling corresponds to cross-frequency coupling where signals from higher-order observer windows orchestrate the activity of lower-order observer windows. Information traveling up the nested hierarchy builds in abstraction whereas top-down signals submerge to disseminate control. Simple thoughts, like the desire to swing a tennis racket, activate a complex system of muscle activations. Higher-order observer windows have successively limited access to lower-order windows, which explains why the intent to act is sometimes met with resistance and optical illusions persist despite knowledge to the contrary.

Observer windows, formed by synchrony, are the sole means of binding information into a unified representation. Synchrony sets an intrinsic processing speed for each observer window that determines the flow of time within the observer window. The brain possesses a wide array of spatial scales with slower rates in higher-order brain nuclei and faster rates in lower-order neurons. Within a given scale, observer windows create unique vantage points with differential access to information. In everyday experience, we might for example focus on driving a car—processing a fast-paced environment on a busy freeway, then slip into abstract thought as minutes pass quickly by, only to re-engage with driving when required to exit the freeway.

Observer windows engage in a dynamic dialogue through coherence. Akin to a conversation, observer windows maintain self-autonomy while communicating a distinct account of events and advocating for often competing action plans. Dialogue through coherence explains how conflicting worldviews can be entertained and paralyzing decision-making is a commonplace occurrence. Dynamic functional networks of observer windows are akin to a corporation working towards a common goal. Curious states of mind such as DID might arise after trauma when communication between some observer windows is reduced or discontinued.

It is our intention that the NOW Model provides a novel description of mental life from simple principles with testable predictions to guide interdisciplinary investigation into the neural basis of consciousness. By standardizing approaches with clear terminology within a single framework, it is our hope that researchers will utilize these definitions to facilitate scientific communication. While the broad scope of the NOW Model requires a diverse range of expertise, we believed it helpful to propose an evocative, yet simple, model that could serve as an anchor point for debate and refinement. Even if elements of our theory turn out to be inaccurate upon new evidence, it is our conviction that the general principles will nevertheless serve as a framework for future inquiry. Finally, the NOW Model provides a convenient way to compare and potentially integrate different theories of consciousness.

## Data Availability

There was no original data generated for this paper.

## References

[R1] Agarwal G, Stevenson IH, Berényi A et al. Spatially distributed local fields in the hippocampus encode rat position. *Science* 2014;344:626–30. 24812401 10.1126/science.1250444 PMC4909490

[R2] Alagapan S, Riddle J, Huang WA et al. Network-targeted, multi-site direct cortical stimulation enhances working memory by modulating phase lag of low-frequency oscillations. *Cell Rep* 2019;29:2590–2598. e2594. 31775030 10.1016/j.celrep.2019.10.072 PMC6901101

[R3] Albouy P, Weiss A, Baillet S et al. Selective entrainment of theta oscillations in the dorsal stream causally enhances auditory working memory performance. *Neuron* 2017;94:193–206. e195. 28343866 10.1016/j.neuron.2017.03.015

[R4] Alekseichuk I, Turi Z, De Lara GA et al. Spatial working memory in humans depends on theta and high gamma synchronization in the prefrontal cortex. *Curr Biol* 2016;26:1513–21. 27238283 10.1016/j.cub.2016.04.035

[R5] Anastassiou CA, Perin R, Markram H et al. Ephaptic coupling of cortical neurons. *Nat Neurosci* 2011;14:217–3. 21240273 10.1038/nn.2727

[R6] Arnal LH, Giraud A-L. Cortical oscillations and sensory predictions. *Trends Cogn Sci* 2012;16:390–8. 22682813 10.1016/j.tics.2012.05.003

[R7] Atasoy S, Deco G, Kringelbach ML et al. Harmonic brain modes: a unifying framework for linking space and time in brain dynamics. *Neuroscientist* 2018;24:277–93. 28863720 10.1177/1073858417728032

[R8] Baars BJ . In the theatre of consciousness. Global workspace theory, a rigorous scientific theory of consciousness. *J Conscious Stud* 1997;4:292–309.

[R9] Baars BJ . Global workspace theory of consciousness: toward a cognitive neuroscience of human experience. *Prog Brain Res* 2005;150:45–53. 16186014 10.1016/S0079-6123(05)50004-9

[R10] Baars BJ, Franklin S. An architectural model of conscious and unconscious brain functions: global workspace theory and IDA. *Neural Netw* 2007;20:955–61. 17998071 10.1016/j.neunet.2007.09.013

[R11] Backus AR, Schoffelen J-M, Szebényi S et al. Hippocampal-prefrontal theta oscillations support memory integration. *Cur Bio* 2016;26:450–7. 10.1016/j.cub.2015.12.048 26832442

[R12] Badre D, Nee DE. Frontal cortex and the hierarchical control of behavior. *Trends Cogn Sci* 2018;22:170–88. 29229206 10.1016/j.tics.2017.11.005 PMC5841250

[R13] Barrett D . Dreams in dissociative disorders. *Dreaming* 1994;4:165–75.

[R14] Bartels A, Zeki S. The theory of multistage integration in the visual brain. *Proc R Soc B: Biol Sci* 1998;265:2327–32. 10.1098/rspb.1998.0579 PMC1689532 9881478

[R15] Bassett DS, Sporns O. Network neuroscience. *Nat Neurosci* 2017;20:353–64. 28230844 10.1038/nn.4502 PMC5485642

[R16] Bedard C, Kroeger H, Destexhe A. Does the 1/f frequency scaling of brain signals reflect self-organized critical states? *Phys Rev Lett* 2006;97:118102. 10.1103/PhysRevLett.97.118102 17025932

[R17] Bernstein A, Hadash Y, Fresco DM. Metacognitive processes model of decentering: emerging methods and insights. *Curr Opini Psychol* 2019;28:245–51. 10.1016/j.copsyc.2019.01.019 PMC7459352 30908987

[R18] Berridge MJ . Neuronal calcium signaling. *Neuron* 1998;21:13–26. 9697848 10.1016/s0896-6273(00)80510-3

[R19] Bertolero MA, Yeo BT, Bassett DS et al. A mechanistic model of connector hubs, modularity and cognition. *Nat Hum Behav* 2018;2:765–77. 30631825 10.1038/s41562-018-0420-6 PMC6322416

[R20] Bertolero MA, Yeo BT, D’Esposito M. The modular and integrative functional architecture of the human brain. *Proc Natl Acad Sci* 2015;112:E6798–807. 26598686 10.1073/pnas.1510619112 PMC4679040

[R21] Bokil H, Laaris N, Blinder K et al. Ephaptic interactions in the mammalian olfactory system. *J Neurosci* 2001;21:RC173–RC173. 11588203 10.1523/JNEUROSCI.21-20-j0004.2001 PMC6763860

[R22] Bowman GR, Pande VS. Protein folded states are kinetic hubs. *Proc Natl Acad Sci* 2010;107:10890–5. 20534497 10.1073/pnas.1003962107 PMC2890711

[R23] Bruce C, Desimone R, Gross CG. Visual properties of neurons in a polysensory area in superior temporal sulcus of the macaque. *J Neurophysiol* 1981;46:369–84. 6267219 10.1152/jn.1981.46.2.369

[R24] Brun VH, Otnæss MK, Molden S et al. Place cells and place recognition maintained by direct entorhinal-hippocampal circuitry. *Science* 2002;296:2243–6. 12077421 10.1126/science.1071089

[R25] Bunde A Havlin S . A brief introduction to fractal geometry. In: Bunde A, Havlin S (eds.), *Fractals in Science*. New York, NY: Springer, 1994, 1–26.

[R26] Buschman TJ, Miller EK. Top-down versus bottom-up control of attention in the prefrontal and posterior parietal cortices. *Science* 2007;315:1860–2. 17395832 10.1126/science.1138071

[R27] Buzsaki G . *Rhythms of the Brain*. Oxford, UK: Oxford University Press, 2006.

[R28] Buzsáki G . Theta oscillations in the hippocampus. *Neuron* 2002;33:325–40. 11832222 10.1016/s0896-6273(02)00586-x

[R29] Buzsáki G, Anastassiou CA, Koch C. The origin of extracellular fields and currents—EEG, ECoG, LFP and spikes. *Nat Rev Neurosci* 2012;13:407–20. 22595786 10.1038/nrn3241 PMC4907333

[R30] Callaway E, Yeager CL. Relationship between reaction time and electroencephalographic alpha phase. *Science* 1960;132:1765–6. 13689987 10.1126/science.132.3441.1765

[R31] Cancela JM, Van Coppenolle F, Galione A et al. Transformation of local Ca2+ spikes to global Ca2+ transients: the combinatorial roles of multiple Ca2+ releasing messengers. *EMBO J* 2002;21:909–19. 11867519 10.1093/emboj/21.5.909 PMC125894

[R32] Canolty RT, Knight RT. The functional role of cross-frequency coupling. *Trends Cogn Sci* 2010;14:506–15. 20932795 10.1016/j.tics.2010.09.001 PMC3359652

[R33] Chalmers D . Panpsychism and panprotopsychism. *Conscious Phy Wor: Perspec Russell Monis* 2015;246:246–76.

[R34] Christensen EM . The online community: DID and plurality. *Eur J Trauma Dissociation* 2022;6:100257.

[R35] Christoff K, Gordon AM, Smallwood J et al. Experience sampling during fMRI reveals default network and executive system contributions to mind wandering. *Proc Nat Acad Sci* 2009;106:8719–24. 19433790 10.1073/pnas.0900234106 PMC2689035

[R36] Chung HS, McHale K, Louis JM et al. Single-molecule fluorescence experiments determine protein folding transition path times. *Science* 2012;335:981–4. 22363011 10.1126/science.1215768 PMC3878298

[R37] Clewett D, DuBrow S, Davachi L. Transcending time in the brain: how event memories are constructed from experience. *Hippocampus* 2019;29:162–83. 30734391 10.1002/hipo.23074 PMC6629464

[R38] Clouter A, Shapiro KL, Hanslmayr S. Theta phase synchronization is the glue that binds human associative memory. *Curr Biol* 2017;27:3143–3148. e3146. 28988860 10.1016/j.cub.2017.09.001

[R39] Cochrane T . A case of shared consciousness. *Synthese* 2021;199:1019–37.

[R40] Cole S, Voytek B. Cycle-by-cycle analysis of neural oscillations. *J Neurophysiol* 2019;122:849–61. 31268801 10.1152/jn.00273.2019

[R41] Colgin LL, Denninger T, Fyhn M et al. Frequency of gamma oscillations routes flow of information in the hippocampus. *Nature* 2009;462:353–7. 19924214 10.1038/nature08573

[R42] Collins AG, Frank MJ. Cognitive control over learning: creating, clustering, and generalizing task-set structure. *Psychol Rev* 2013;120:190. 10.1037/a0030852 PMC3974273 23356780

[R43] Császár N, Scholkmann F, Kapócs G et al. The “hidden observer” as the cognitive unconscious during hypnosis. *Act Nerv Super* 2016;58:51–61.

[R44] Csikszentmihalyi M . *Applications of Flow in Human Development and Education*. New York, NY: Springer, 2014.

[R45] Dehaene S, Kerszberg M, Changeux J-P. A neuronal model of a global workspace in effortful cognitive tasks. *Proc Natl Acad Sci* 1998;95:14529–34. 9826734 10.1073/pnas.95.24.14529 PMC24407

[R46] Dennett DC . Quining qualia. *Conscious Contemp Sci* 1988;42–77.

[R47] Dennett DC . *Consciousness Explained*. Boston, MA: Little, Brown and Co., 1991.

[R48] Dennett DC, Kinsbourne M. Time and the observer: the where and when of consciousness in the brain. *Behav Brain Sci* 1992;15:183–201.

[R49] Dikker S, Wan L, Davidesco I et al. Brain-to-brain synchrony tracks real-world dynamic group interactions in the classroom. *Curr Biol* 2017;27:1375–80. 28457867 10.1016/j.cub.2017.04.002

[R50] Donoghue T, Haller M, Peterson EJ et al. Parameterizing neural power spectra into periodic and aperiodic components. *Nat Neurosci* 2020;23:1655–65. 33230329 10.1038/s41593-020-00744-x PMC8106550

[R51] Dorahy MJ, Brand BL, Şar V et al. Dissociative identity disorder: an empirical overview. *Aust N Z J Psychiatry* 2014;48:402–17. 24788904 10.1177/0004867414527523

[R52] Duff EP, Johnston LA, Xiong J et al. The power of spectral density analysis for mapping endogenous BOLD signal fluctuations. *Hum Brain Mapp* 2008;29:778–90. 18454458 10.1002/hbm.20601 PMC5441229

[R53] Dunne JD, Thompson E, Schooler J. Mindful meta-awareness: sustained and non-propositional. *Curr Opin Psychol* 2019;28:307–11. 31374535 10.1016/j.copsyc.2019.07.003

[R54] Edelman DB, Baars BJ, Seth AK. Identifying hallmarks of consciousness in non-mammalian species. *Conscious Cogn* 2005;14:169–87. 15766896 10.1016/j.concog.2004.09.001

[R55] Edelman GM, Gally JA. Reentry: a key mechanism for integration of brain function. *Front Integr Neurosci* 2013;63. 10.3389/fnint.2013.00063 PMC3753453 23986665

[R56] Engel AK, Singer W. Temporal binding and the neural correlates of sensory awareness. *Trends Cogn Sci* 2001;5:16–25. 11164732 10.1016/s1364-6613(00)01568-0

[R57] Fanelli D . A theory and methodology to quantify knowledge. *R Soc Open Sci* 2019;6:181055. 10.1098/rsos.181055 PMC6502358 31183113

[R58] Felleman DJ, Van Essen DC. Distributed hierarchical processing in the primate cerebral cortex. *Cereb Cortex* 1991;1:1–47. 1822724 10.1093/cercor/1.1.1-a

[R59] Fiebelkorn IC, Kastner S. A Rhythmic Theory of Attention. *Trends Cogn Sci* 2018;23:87–101. 30591373 10.1016/j.tics.2018.11.009 PMC6343831

[R60] Fiebelkorn IC, Pinsk MA, Kastner S. A dynamic interplay within the frontoparietal network underlies rhythmic spatial attention. *Neuron* 2018;99:842–853. e848. 30138590 10.1016/j.neuron.2018.07.038 PMC6474777

[R61] Fisher MP . Quantum cognition: the possibility of processing with nuclear spins in the brain. *Ann Phys* 2015;362:593–602.

[R62] Flegal KE, Anderson MC. Overthinking skilled motor performance: or why those who teach can’t do. *Psychon Bull Rev* 2008;15:927–32. 18926983 10.3758/PBR.15.5.927

[R63] Fodor JA . *The Modularity of Mind*. Cambridge, MA: MIT Press, 1983.

[R64] Frederick KK, Marlow MS, Valentine KG et al. Conformational entropy in molecular recognition by proteins. *Nature* 2007;448:325–9. 17637663 10.1038/nature05959 PMC4156320

[R65] Freeman WJ . Mechanism and significance of global coherence in scalp EEG. *Curr Opin Neurobiol* 2015;31:199–205. 25506772 10.1016/j.conb.2014.11.008

[R66] Fries P . A mechanism for cognitive dynamics: neuronal communication through neuronal coherence. *Trends Cogn Sci* 2005;9:474–80. 16150631 10.1016/j.tics.2005.08.011

[R67] Fries P . Rhythms for cognition: communication through coherence. *Neuron* 2015;88:220–35. 26447583 10.1016/j.neuron.2015.09.034 PMC4605134

[R68] Fries P, Reynolds JH, Rorie AE et al. Modulation of oscillatory neuronal synchronization by selective visual attention. *Science* 2001;291:1560–3. 11222864 10.1126/science.1055465

[R69] Friston K . The free-energy principle: a unified brain theory? *Nat Rev Neurosci* 2010;11:127–38. 20068583 10.1038/nrn2787

[R70] Fröhlich F, McCormick DA. Endogenous electric fields may guide neocortical network activity. *Neuron* 2010;67:129–43. 20624597 10.1016/j.neuron.2010.06.005 PMC3139922

[R71] Gable SL, Hopper EA, Schooler JW. When the muses strike: creative ideas of physicists and writers routinely occur during mind wandering. *Psychol Sci* 2019;30:396–404. 30653407 10.1177/0956797618820626

[R72] Gallagher HL, Frith CD. Functional imaging of ‘theory of mind’. *Trends Cogn Sci* 2003;7:77–83. 12584026 10.1016/s1364-6613(02)00025-6

[R73] Gallen CL, D’Esposito M. Brain modularity: a biomarker of intervention-related plasticity. *Trends Cogn Sci* 2019;23:293–304. 30827796 10.1016/j.tics.2019.01.014 PMC6750199

[R74] Gazzaniga MS . Principles of human brain organization derived from split-brain studies. *Neuron* 1995;14:217–28. 7857634 10.1016/0896-6273(95)90280-5

[R75] Gazzaniga MS . Cerebral specialization and interhemispheric communication: does the corpus callosum enable the human condition? *Brain* 2000;123:1293–326. 10869045 10.1093/brain/123.7.1293

[R76] Gazzaniga MS . Forty-five years of split-brain research and still going strong. *Nat Rev Neurosci* 2005;6:653–9. 16062172 10.1038/nrn1723

[R77] Gentet LJ, Stuart GJ, Clements JD. Direct measurement of specific membrane capacitance in neurons. *Biophys J* 2000;79:314–20. 10866957 10.1016/S0006-3495(00)76293-X PMC1300935

[R78] Ghitza O . The theta-syllable: a unit of speech information defined by cortical function. *Front Psychol* 2013;4:138. 10.3389/fpsyg.2013.00138 PMC3602725 23519170

[R79] Ghosh A, Greenberg ME. Calcium signaling in neurons: molecular mechanisms and cellular consequences. *Science* 1995;268:239–47. 7716515 10.1126/science.7716515

[R80] Godfrey-Smith P . *Other Minds: The Octopus, the Sea, and the Deep Origins of Consciousness*. New York, NY: Farrar, Straus and Giroux, 2016.

[R81] Goetze T, Brickmann J. Self similarity of protein surfaces. *Biophys J* 1992;61:109–18. 1540684 10.1016/S0006-3495(92)81820-9 PMC1260227

[R82] Goff P . VI—Panpsychism and Free Will: A Case Study in Liberal Naturalism. *Proceedings of the Aristotelian Society* 2020;120:123–44.

[R83] Goff P Coleman S . Russellian monism. In: Kriegel U (ed.), *The Oxford Handbook of the Philosophy of Consciousness* Oxford, UK: Oxford University Press, 2020, 301–27.

[R84] Goodenough DA, Paul DL. Gap junctions. *Cold Spring Harb perspect biol* 2009;1:a002576. 10.1101/cshperspect.a002576 PMC2742079 20066080

[R85] Gray JR, Chabris CF, Braver TS. Neural mechanisms of general fluid intelligence. *Nat Neurosci* 2003;6:316–22. 12592404 10.1038/nn1014

[R86] Hagura N, Kanai R, Orgs G et al. Ready steady slow: action preparation slows the subjective passage of time. *Proc R Soc B: Biol Sci* 2012;279:4399–406. 10.1098/rspb.2012.1339 PMC3479796 22951740

[R87] Hanslmayr S, Gross J, Klimesch W et al. The role of alpha oscillations in temporal attention. *Brain Res Rev* 2011;67:331–43. 21592583 10.1016/j.brainresrev.2011.04.002

[R88] Harmon-Jones E Mills J . An introduction to cognitive dissonance theory and an overview of current perspectives on the theory. 2nd In: Harmon-Jones E (ed.), *Cognitive dissonance: Reexamining a pivotal theory in psychology*. Washington, DC, US: American Psychological Association, 2019, 3–24.

[R89] He BJ . Scale-free brain activity: past, present, and future. *Trends Cogn Sci* 2014;18:480–7. 24788139 10.1016/j.tics.2014.04.003 PMC4149861

[R90] Helfrich RF, Fiebelkorn IC, Szczepanski SM et al.. Neural mechanisms of sustained attention are rhythmic. *Neuron* 2018;99:854–865. e855. 30138591 10.1016/j.neuron.2018.07.032 PMC6286091

[R91] Helfrich RF, Huang M, Wilson G et al. Prefrontal cortex modulates posterior alpha oscillations during top-down guided visual perception. *Proc Natl Acad Sci* 2017;114:9457–62. 28808023 10.1073/pnas.1705965114 PMC5584435

[R92] He BJ, Raichle ME. The fMRI signal, slow cortical potential and consciousness. *Trends Cogn Sci* 2009;13:302–9. 19535283 10.1016/j.tics.2009.04.004 PMC2855786

[R93] Hermiller MS, Chen YF, Parrish TB et al. Evidence for immediate enhancement of hippocampal memory encoding by network-targeted theta-burst stimulation during concurrent fMRI. *J Neurosci* 2020;40:7155–68. 32817326 10.1523/JNEUROSCI.0486-20.2020 PMC7480242

[R94] Herzog MH, Kammer T, Scharnowski F. Time slices: what is the duration of a percept? *PLoS Biol* 2016;14:e1002433. 10.1371/journal.pbio.1002433 PMC4829156 27070777

[R95] He BJ, Snyder AZ, Zempel JM et al. Electrophysiological correlates of the brain’s intrinsic large-scale functional architecture. *Proc Natl Acad Sci* 2008;105:16039–44. 18843113 10.1073/pnas.0807010105 PMC2564983

[R96] Hilgard ER . Divided consciousness in hypnosis: The implications of the hidden observer. In: Shor RE (ed.), *Hypnosis*. New York, NY: Routledge, 2017, 45–80.

[R97] Hines ML, Carnevale NT. The NEURON simulation environment. *Neural Computation* 1997;9:1179–209. 9248061 10.1162/neco.1997.9.6.1179

[R98] Hoffman KL, Dragan MC, Leonard TK et al. Saccades during visual exploration align hippocampal 3–8 Hz rhythms in human and non-human primates. *Front Syst Neurosci* 2013;7:43. 10.3389/fnsys.2013.00043 PMC3757337 24009562

[R99] Hoffman DD, Singh M, Prakash C. The interface theory of perception. *Psychon Bull Rev* 2015;22:1480–506. 26384988 10.3758/s13423-015-0890-8

[R100] Hofstadter DR . *I Am a Strange Loop*. New York, NY: Basic books, 2007.

[R101] Hunt T, Schooler JW. The easy part of the hard problem: a resonance theory of consciousness. *Front Syst Neurosci* 2019;13:378. 10.3389/fnhum.2019.00378 PMC6834646 31736728

[R102] Ibrahim LA, Schuman B, Bandler R et al. Mining the jewels of the cortex’s crowning mystery. *Curr Opin Neurobiol* 2020;63:154–61. 32480351 10.1016/j.conb.2020.04.005 PMC8075042

[R103] Ingram D . *Mastering the Core Teachings of the Buddha: An Unusually Hardcore Dharma Book-Revised and Expanded Edition*. Newburyport, MA: Red Wheel/Weiser, 2018.

[R104] Ivanov PC, Amaral LAN, Goldberger AL et al. Multifractality in human heartbeat dynamics. *Nature* 1999;399:4615. 10.1038/20924 10365957

[R105] James W . *The Principles of Psychology*. New York, NY: Henry Holt and Company, 1890.

[R106] Jaušovec N, Jaušovec K. Increasing working memory capacity with theta transcranial alternating current stimulation (tACS). *Biol Psychol* 2014;96:42–7. 24291565 10.1016/j.biopsycho.2013.11.006

[R107] Jensen O, Colgin LL. Cross-frequency coupling between neuronal oscillations. *Trends Cogn Sci* 2007;11:267–9. 17548233 10.1016/j.tics.2007.05.003

[R108] Kastrup B . The universe in consciousness. *J Conscious Stud* 2018;25:125–55.

[R109] Khader P, Schicke T, Röder B et al. On the relationship between slow cortical potentials and BOLD signal changes in humans. *Int J Psychophysiol* 2008;67:252–61. 17669531 10.1016/j.ijpsycho.2007.05.018

[R110] Klimesch W . An algorithm for the EEG frequency architecture of consciousness and brain body coupling. *Front Hum Neurosci* 2013;7:766. 10.3389/fnhum.2013.00766 PMC3824085 24273507

[R111] Klimesch W . The frequency architecture of brain and brain body oscillations: an analysis. *Eur J Neurosci* 2018;48:2431–53. 30281858 10.1111/ejn.14192 PMC6668003

[R112] Klimesch W . Heartbeat, brain oscillations and body awareness: a commentary. *J Integr Neurosci* 2023;22:155. 10.31083/j.jin2206155 38176946

[R113] Koch C, Massimini M, Boly M et al. Neural correlates of consciousness: progress and problems. *Nat Rev Neurosci* 2016;17:307–21. 27094080 10.1038/nrn.2016.22

[R114] Kornmeier J, Friedel E, Wittmann M et al. EEG correlates of cognitive time scales in the Necker-Zeno model for bistable perception. *Conscious Cogn* 2017;53:136–50. 28666186 10.1016/j.concog.2017.04.011

[R115] Kornmeier J, Hein CM, Bach M. Multistable perception: when bottom-up and top-down coincide. *Brain Cogn* 2009;69:138–47. 18682314 10.1016/j.bandc.2008.06.005

[R116] Lau H, Rosenthal D. Empirical support for higher-order theories of conscious awareness. *Trends Cogn Sci* 2011;15:365–73. 21737339 10.1016/j.tics.2011.05.009

[R117] Lawrie SM, Buechel C, Whalley HC et al. Reduced frontotemporal functional connectivity in schizophrenia associated with auditory hallucinations. *Biol Psychiatry* 2002;51:1008–11. 12062886 10.1016/s0006-3223(02)01316-1

[R118] Levin M, Martyniuk CJ. The bioelectric code: an ancient computational medium for dynamic control of growth and form. *Biosystems* 2018;164:76–93. 28855098 10.1016/j.biosystems.2017.08.009 PMC10464596

[R119] Lisman JE, Jensen O. The theta-gamma neural code. *Neuron* 2013;77:1002–16. 23522038 10.1016/j.neuron.2013.03.007 PMC3648857

[R120] Liu X, Han F, Fu R et al. Epileptogenic zone location of temporal lobe epilepsy by cross-frequency coupling analysis. *Front Neurol* 2021;12:764821. 10.3389/fneur.2021.764821 PMC8636749 34867749

[R121] Loftus EF, Schooler JW. Information processing conceptualizations. *Inf Behav* 1985;225–50.

[R122] Luria AR . *Higher Cortical Functions in Man*. New York, NY: Springer, 2012.

[R123] Mashour GA, Roelfsema P, Changeux J-P et al. Conscious processing and the global neuronal workspace hypothesis. *Neuron* 2020;105:776–98. 32135090 10.1016/j.neuron.2020.01.026 PMC8770991

[R124] Metzinger T . *The Ego Tunnel: The Science of the Mind and the Myth of the Self*. New York, NY: Basic Books, 2009.

[R125] Metzinger T . The Problem of Mental Action. In: Metzinger T, Wiese W (eds.), *Philosophy and Predictive Processing*. Frankfurt am Main, Germany: MIND Group, 2017;19:1–26.

[R126] Minsky M . *Society of Mind*. New York, NY: Simon and Schuster, 1988.

[R127] Monto S, Palva S, Voipio J et al. Very slow EEG fluctuations predict the dynamics of stimulus detection and oscillation amplitudes in humans. *J Neurosci* 2008;28:8268–72. 18701689 10.1523/JNEUROSCI.1910-08.2008 PMC6670577

[R128] Morillon B, Arnal LH, Schroeder CE et al. Prominence of delta oscillatory rhythms in the motor cortex and their relevance for auditory and speech perception. *Neurosci Biobehav Rev* 2019;107:136–42. 31518638 10.1016/j.neubiorev.2019.09.012

[R129] Mori H, Mishina M. Structure and function of the NMDA receptor channel. *Neuropharmacology* 1995;34:1219–37. 8570021 10.1016/0028-3908(95)00109-j

[R130] Murray JD, Bernacchia A, Freedman DJ et al. A hierarchy of intrinsic timescales across primate cortex. *Nat Neurosci* 2014;17:1661–3. 25383900 10.1038/nn.3862 PMC4241138

[R131] Nee DE, D’Esposito M. The hierarchical organization of the lateral prefrontal cortex. *Elife* 2016;5:e12112. 10.7554/eLife.12112 PMC4811776 26999822

[R132] Northoff G . Paradox of slow frequencies”–are slow frequencies in upper cortical layers a neural predisposition of the level/state of consciousness (NPC)?. *Conscious Cogn* 2017;54:20–35. 28392004 10.1016/j.concog.2017.03.006

[R133] Northoff G, Huang Z. How do the brain’s time and space mediate consciousness and its different dimensions? Temporo-spatial theory of consciousness (TTC). *Neurosci Biobehav Rev* 2017;80:630–45. 28760626 10.1016/j.neubiorev.2017.07.013

[R134] Nunez PL, Srinivasan R. A theoretical basis for standing and traveling brain waves measured with human EEG with implications for an integrated consciousness. *Clin Neurophysiol* 2006;117:2424–35. 16996303 10.1016/j.clinph.2006.06.754 PMC1991284

[R135] O’Keefe J, Recce ML. Phase relationship between hippocampal place units and the EEG theta rhythm. *Hippocampus* 1993;3:317–30. 8353611 10.1002/hipo.450030307

[R136] O’shea H, Moran A. Are fast complex movements unimaginable? Pupillometric studies of motor imagery in expert piano playing. *J Mot Behav* 2018;51:371–84. 30277448 10.1080/00222895.2018.1485010

[R137] Palva JM, Palva S. Functional integration across oscillation frequencies by cross‐frequency phase synchronization. *Eur J Neurosci* 2018;48:2399–406. 29094462 10.1111/ejn.13767

[R138] Pang JC, Aquino KM, Oldehinkel M et al. Geometric constraints on human brain function. *Nature* 2023;618:566–74. 37258669 10.1038/s41586-023-06098-1 PMC10266981

[R139] Park H, Ince RA, Schyns PG et al. Frontal top-down signals increase coupling of auditory low-frequency oscillations to continuous speech in human listeners. *Curr Biol* 2015;25:1649–53. 26028433 10.1016/j.cub.2015.04.049 PMC4503802

[R140] Parkinson C, Kleinbaum AM, Wheatley T. Similar neural responses predict friendship. *Nat Commun* 2018;9:1–14. 29382820 10.1038/s41467-017-02722-7 PMC5790806

[R141] Peelle JE, Gross J, Davis MH. Phase-locked responses to speech in human auditory cortex are enhanced during comprehension. *Cereb Cortex* 2013;23:1378–87. 22610394 10.1093/cercor/bhs118 PMC3643716

[R142] Pinotsis DA, Fridman G, Miller EK. Cytoelectric coupling: electric fields sculpt neural activity and “tune” the brain’s infrastructure. *Prog Neurobiol* 2023;226:102465. 10.1016/j.pneurobio.2023.102465 PMC12302380 37210066

[R143] Platchias D . *Phenomenal Consciousness: Understanding the Relation between Experience and Neural Processes in the Brain*. London, UK: Routledge, 2014.

[R144] Poeppel D, Assaneo MF. Speech rhythms and their neural foundations. *Nat Rev Neurosci* 2020;21:1–13. 32376899 10.1038/s41583-020-0304-4

[R145] Polanía R, Nitsche MA, Korman C et al. The importance of timing in segregated theta phase-coupling for cognitive performance. *Curr Biol* 2012;22:1314–8. 22683259 10.1016/j.cub.2012.05.021

[R146] Raymond JE, Shapiro KL, Arnell KM. Temporary suppression of visual processing in an RSVP task: an attentional blink? *J Exp Psychol Hum Percept Perform* 1992;18:849. 10.1037//0096-1523.18.3.849 1500880

[R147] Reichle ED, Reineberg AE, Schooler JW. Eye movements during mindless reading. *Psychol Sci* 2010;21:1300–10. 20679524 10.1177/0956797610378686

[R148] Riddle J, Alexander ML, Schiller CE et al. Reward-based decision-making engages distinct modes of cross-frequency coupling. *Cereb Cort* 2022;32:2079–94. 10.1093/cercor/bhab336 PMC9113280 34622271

[R149] Riddle J, Frohlich F. Targeting neural oscillations with transcranial alternating current stimulation. *Brain Res* 2021;1765:147491. 10.1016/j.brainres.2021.147491 PMC8206031 33887251

[R150] Riddle J, McFerren A, Frohlich F. Causal role of cross-frequency coupling in distinct components of cognitive control. *Prog Neurobiol* 2021;202:102033. 10.1016/j.pneurobio.2021.102033 PMC8184612 33741402

[R151] Riddle J, Scimeca JM, Cellier D et al. Causal evidence for a role of theta and alpha oscillations in the control of working memory. *Curr Biol* 2020a;30:1748–1754.e4. 32275881 10.1016/j.cub.2020.02.065 PMC7202975

[R152] Riddle J, Vogelsang DA, Hwang K et al. Distinct oscillatory dynamics underlie different components of hierarchical cognitive control. *J Neurosci* 2020b;40:4945–53. 32430297 10.1523/JNEUROSCI.0617-20.2020 PMC7326361

[R153] Riesenhuber M, Poggio T. Hierarchical models of object recognition in cortex. *Nat Neurosci* 1999;2:1019–25. 10526343 10.1038/14819

[R154] Rizzuto R, Pozzan T. Microdomains of intracellular Ca2+: molecular determinants and functional consequences. *Physiol Rev* 2006;86:369–408. 16371601 10.1152/physrev.00004.2005

[R155] Romei V, Thut G, Silvanto J. Information-based approaches of noninvasive transcranial brain stimulation. *Trends Neurosci* 2016;39:782–95. 27697295 10.1016/j.tins.2016.09.001

[R156] Rosenthal DM . The independence of consciousness and sensory quality. *Philos Issues* 1991;1:15–36.

[R157] Rosenthal D . Thinking that one thinks. *Language Thought* 1993;259–87.

[R158] Saalmann YB, Pinsk MA, Wang L et al. The pulvinar regulates information transmission between cortical areas based on attention demands. *Science* 2012;337:753–6. 22879517 10.1126/science.1223082 PMC3714098

[R159] Samaha J, Postle BR. The speed of alpha-band oscillations predicts the temporal resolution of visual perception. *Curr Biol* 2015;25:2985–90. 26526370 10.1016/j.cub.2015.10.007 PMC4654641

[R160] Sauer S, Lemke J, Wittmann M et al. How long is now for mindfulness meditators? *Pers Individ Differ* 2012;52:750–4.

[R161] Sauseng P, Klimesch W, Heise KF et al. Brain oscillatory substrates of visual short-term memory capacity. *Current Biology* 2009;19:1846–52. 19913428 10.1016/j.cub.2009.08.062

[R162] Schlumpf YR, Reinders AA, Nijenhuis ER et al. Dissociative part-dependent resting-state activity in dissociative identity disorder: a controlled fMRI perfusion study. *PLoS One* 2014;9:e98795. 10.1371/journal.pone.0098795 PMC4055615 24922512

[R163] Schmidhuber J . 2008. Driven by compression progress: a simple principle explains essential aspects of subjective beauty, novelty, surprise, interestingness, attention, curiosity, creativity, art, science, music, jokes. *Paper presented at the Workshop on Anticipatory Behavior In Adaptive Learning Systems*, Zagreb, Croatia, September, 2008;12:3–5.

[R164] Schooler JW . Re-representing consciousness: dissociations between experience and meta-consciousness. *Trends Cogn Sci* 2002;6:339–44. 12140084 10.1016/s1364-6613(02)01949-6

[R165] Schooler JW . Zoning out while reading: evidence for dissociations between experience and metaconsciousness Jonathan W. Schooler, Erik D. Reichle, and David V. Halpern. *Thinking Seeing* 2004;203:1942–7.

[R166] Schooler J . Bridging the objective/subjective divide: towards a meta-perspective of science and experience. Open MIND. Frankfurt am Main: MIND Group, 2014.

[R167] Schooler JW, Hunt T, Schooler JN. Reconsidering the metaphysics of science from the inside out *Neuroscience, Consciousness and Spirituality*. Springer, 157–94, 2011a.

[R168] Schooler JW, Smallwood J, Christoff K et al. Meta-awareness, perceptual decoupling and the wandering mind. *Trends Cogn Sci* 2011b;15:319–26. 21684189 10.1016/j.tics.2011.05.006

[R169] Schott BH, Wüstenberg T, Wimber M et al.. The relationship between level of processing and hippocampal–cortical functional connectivity during episodic memory formation in humans. *Hum Brain Mapp* 2013;34:407–24. 22042493 10.1002/hbm.21435 PMC6870091

[R170] Schwartz RC, Sweezy M. *Internal Family Systems Therapy*. New York, NY: Guilford Press, 2019.

[R171] Serences JT, Yantis S. Selective visual attention and perceptual coherence. *Trends Cogn Sci* 2006;10:38–45. 16318922 10.1016/j.tics.2005.11.008

[R172] Seth AK, Suzuki K, Critchley HD. An interoceptive predictive coding model of conscious presence. *Front Psychol* 2012;2:395. 10.3389/fpsyg.2011.00395 PMC3254200 22291673

[R173] Shapiro KL, Raymond J, Arnell K. The attentional blink. *Trends Cogn Sci* 1997;1:291–6. 21223931 10.1016/S1364-6613(97)01094-2

[R174] Silver MA, Kastner S. Topographic maps in human frontal and parietal cortex. *Trends Cogn Sci* 2009;13:488–95. 19758835 10.1016/j.tics.2009.08.005 PMC2767426

[R175] Singer W, Gray CM. Visual feature integration and the temporal correlation hypothesis. *Ann Rev Neurosci* 1995;18:555–86. 7605074 10.1146/annurev.ne.18.030195.003011

[R176] Smallwood J, Schooler JW. The science of mind wandering: empirically navigating the stream of consciousness. *Ann Rev Psychol* 2015;66:487–518. 25293689 10.1146/annurev-psych-010814-015331

[R177] Smetters D, Majewska A, Yuste R. Detecting action potentials in neuronal populations with calcium imaging. *Methods* 1999;18:215–21. 10356353 10.1006/meth.1999.0774

[R178] Smock RG, Gierasch LM. Sending signals dynamically. *Science* 2009;324:198–203. 19359576 10.1126/science.1169377 PMC2921701

[R179] Sperry R . Consciousness, personal identity and the divided brain. *Neuropsychologia* 1984;22:661–73. 6084824 10.1016/0028-3932(84)90093-9

[R180] Spruston N, Schiller Y, Stuart G et al. Activity-dependent action potential invasion and calcium influx into hippocampal CA1 dendrites. *Science* 1995;268:297–300. 7716524 10.1126/science.7716524

[R181] Stetson C, Fiesta MP, Eagleman DM. Does time really slow down during a frightening event? *PloS One* 2007;2:e1295. 10.1371/journal.pone.0001295 PMC2110887 18074019

[R182] Sweller J . Cognitive load theory. In: *Psychology of Learning and Motivation*. Amsterdam, Netherlands: Elsevier, Vol. 55, 37–76, 2011.

[R183] Taylor P, Hobbs J, Burroni J et al. The global landscape of cognition: hierarchical aggregation as an organizational principle of human cortical networks and functions. *Sci Rep* 2015;5:1–18. 10.1038/srep18112 PMC4681187 26669858

[R184] Teich MC . Fractal character of the auditory neural spike train. *IEEE T Bio-Med Eng* 1989;36:150–60. 10.1109/10.16460 2921061

[R185] Thut G, Veniero D, Romei V et al. Rhythmic TMS causes local entrainment of natural oscillatory signatures. *Curr Biol* 2011;21:1176–85. 21723129 10.1016/j.cub.2011.05.049 PMC3176892

[R186] Toker D, Sommer F. Moving past the minimum information partition: how to quickly and accurately calculate integrated information. *arXiv Preprint arXiv:1605.01096* 2016.

[R187] Tong F, Meng M, Blake R. Neural bases of binocular rivalry. *Trends Cogn Sci* 2006;10:502–11. 16997612 10.1016/j.tics.2006.09.003

[R188] Tononi G, Boly M, Massimini M et al. Integrated information theory: from consciousness to its physical substrate. *Nat Rev Neurosci* 2016;17:450–61. 27225071 10.1038/nrn.2016.44

[R189] Tononi G, Koch C. Consciousness: here, there and everywhere? *Philos Trans R Soc B: Biol Sci* 2015;370:20140167. 10.1098/rstb.2014.0167 PMC4387509 25823865

[R190] Treisman A . The binding problem. *Curr Opin Neurobiol* 1996;6:171–8. 8725958 10.1016/s0959-4388(96)80070-5

[R191] Tsodyks MV, Skaggs WE, Sejnowski TJ et al. Population dynamics and theta rhythm phase precession of hippocampal place cell firing: a spiking neuron model. *Hippocampus* 1996;6:271–80. 8841826 10.1002/(SICI)1098-1063(1996)6:3<271::AID-HIPO5>3.0.CO;2-Q

[R192] Umeton D, Read JC, Rowe C. Unravelling the illusion of flicker fusion. *Biol Lett* 2017;13:20160831. 10.1098/rsbl.2016.0831 PMC5326512 28148834

[R193] Vanhatalo S, Palva JM, Holmes M et al. Infraslow oscillations modulate excitability and interictal epileptic activity in the human cortex during sleep. *Proc Natl Acad Sci* 2004;101:5053–7. 15044698 10.1073/pnas.0305375101 PMC387372

[R194] VanRullen R . Perceptual cycles. *Trends Cogn Sci* 2016;20:723–35. 27567317 10.1016/j.tics.2016.07.006

[R195] VanRullen R, Koch C. Is perception discrete or continuous? *Trends Cogn Sci* 2003;7:207–13. 12757822 10.1016/s1364-6613(03)00095-0

[R196] Van Veen V, Krug MK, Schooler JW et al. Neural activity predicts attitude change in cognitive dissonance. *Nat Neurosci* 2009;12:1469–74. 19759538 10.1038/nn.2413

[R197] Vattay G, Salahub D, Csabai I et al. Quantum criticality at the origin of life. *Paper presented at the Journal of Physics: Conference Series* Castiglioncello, Italy September 15–19, 2014. 2015.

[R198] Venables P, Warwick-Evans L. Cortical arousal and two flash threshold. *Psychon Sci* 1967;8:231–2.

[R199] Vercammen A, Knegtering H, den Boer JA et al. Auditory hallucinations in schizophrenia are associated with reduced functional connectivity of the temporo-parietal area. *Biol Psychiatry* 2010;67:912–8. 20060103 10.1016/j.biopsych.2009.11.017

[R200] Vidyasagar TR, Levichkina E. An integrated neuronal model of claustral function in timing the synchrony between cortical areas. *Front Neural Circuits* 2019;13:3. 10.3389/fncir.2019.00003 PMC6371054 30804759

[R201] Vinck M, Oostenveld R, Van Wingerden M et al. An improved index of phase-synchronization for electrophysiological data in the presence of volume-conduction, noise and sample-size bias. *Neuroimage* 2011;55:1548–65. 21276857 10.1016/j.neuroimage.2011.01.055

[R202] Violante IR, Li LM, Carmichael DW et al. Externally induced frontoparietal synchronization modulates network dynamics and enhances working memory performance. *elife* 2017;6:e22001. 10.7554/eLife.22001 PMC5349849 28288700

[R203] Volz LJ, Gazzaniga MS. Interaction in isolation: 50 years of insights from split-brain research. *Brain* 2017;140:2051–60. 29177496 10.1093/brain/awx139

[R204] Von Stein A, Sarnthein J. Different frequencies for different scales of cortical integration: from local gamma to long range alpha/theta synchronization. *Int J Psychophysiol* 2000;38:301–13. 11102669 10.1016/s0167-8760(00)00172-0

[R205] Voytek B, Kayser AS, Badre D et al. Oscillatory dynamics coordinating human frontal networks in support of goal maintenance. *Nat Neurosci* 2015a;18:1318–24. 26214371 10.1038/nn.4071 PMC4551604

[R206] Voytek B, Kramer MA, Case J et al. Age-related changes in 1/f neural electrophysiological noise. *J Neurosci* 2015b;35:13257–65. 26400953 10.1523/JNEUROSCI.2332-14.2015 PMC4579381

[R207] Wallis G, Stokes M, Cousijn H et al. Frontoparietal and cingulo-opercular networks play dissociable roles in control of working memory. *J Cogn Neurosci* 2015;27:2019–34. 26042457 10.1162/jocn_a_00838

[R208] Wang X-J . Neurophysiological and computational principles of cortical rhythms in cognition. *Physiol Rev* 2010;90:1195–268. 20664082 10.1152/physrev.00035.2008 PMC2923921

[R209] Wang Q, Ng L, Harris JA et al. Organization of the connections between claustrum and cortex in the mouse. *J Comp Neurol* 2017;525:1317–46. 27223051 10.1002/cne.24047 PMC5324679

[R210] Watson BO . Cognitive and physiologic impacts of the infraslow oscillation. *Front Syst Neurosci* 2018;12:44. 10.3389/fnsys.2018.00044 PMC6198276 30386218

[R211] Wegner DM . You can’t always think what you want: problems in the suppression of unwanted thoughts. *Adv Exp Soc Psychol* 1992;25:193–225.

[R212] Wegner DM, Wheatley T. Apparent mental causation: sources of the experience of will. *Am Psychol* 1999;54:480. 10.1037//0003-066x.54.7.480 10424155

[R213] Wei G, Xi W, Nussinov R et al. Protein ensembles: how does nature harness thermodynamic fluctuations for life? The diverse functional roles of conformational ensembles in the cell. *Chem Rev* 2016;116:6516–51. 26807783 10.1021/acs.chemrev.5b00562 PMC6407618

[R214] Wiseman R, Watt C, Gilhooly K et al. Creativity and ease of ambiguous figural reversal. *Br J Psychol* 2011;102:615–22. 21752010 10.1111/j.2044-8295.2011.02031.x

[R215] Woods NM, Cuthbertson KR, Cobbold PH. Repetitive transient rises in cytoplasmic free calcium in hormone-stimulated hepatocytes. *Nature* 1986;319:600–2. 3945348 10.1038/319600a0

[R216] Wutz A, Melcher D, Samaha J. Frequency modulation of neural oscillations according to visual task demands. *Proc Natl Acad Sci* 2018;115:1346–51. 29358390 10.1073/pnas.1713318115 PMC5819398

[R217] Wyart V, de Gardelle V, Scholl J et al. Rhythmic fluctuations in evidence accumulation during decision making in the human brain. *Neuron* 2012;76:847–58. 23177968 10.1016/j.neuron.2012.09.015 PMC3975574

[R218] Zeki S . The disunity of consciousness. *Trends Cogn Sci* 2003;7:214–8. 12757823 10.1016/s1364-6613(03)00081-0

[R219] Zhang J, Huang Z, Chen Y et al. Breakdown in the temporal and spatial organization of spontaneous brain activity during general anesthesia. *Hum Brain Mapp* 2018;39:2035–46. 29377435 10.1002/hbm.23984 PMC6866328

[R220] Zhang B, Song W, Brown J et al. Electronic conductance resonance in non-redox-active proteins. *J Am Chem Soc* 2020;142:6432–8. 32176496 10.1021/jacs.0c01805 PMC7185870

[R221] Zhang B, Song W, Pang P et al. Observation of giant conductance fluctuations in a protein. *Nano Futures* 2017;1:035002. 10.1088/2399-1984/aa8f91 PMC5851656 29552645

